# The NHS England 100,000 Genomes Project: feasibility and utility of centralised genome sequencing for children with cancer

**DOI:** 10.1038/s41416-022-01788-5

**Published:** 2022-04-22

**Authors:** Jamie Trotman, Ruth Armstrong, Helen Firth, Claire Trayers, James Watkins, Kieren Allinson, Thomas S. Jacques, James C. Nicholson, G. A. Amos Burke, Genomics England Research Consortium, J. C. Ambrose, P. Arumugam, R. Bevers, M. Bleda, F. Boardman-Pretty, C. R. Boustred, H. Brittain, M. J. Caulfield, G. C. Chan, T. Fowler, A. Giess, A. Hamblin, S. Henderson, T. J. P. Hubbard, R. Jackson, L. J. Jones, D. Kasperaviciute, M. Kayikci, A. Kousathanas, L. Lahnstein, S. E. A. Leigh, I. U. S. Leong, F. J. Lopez, F. Maleady-Crowe, M. McEntagart, F. Minneci, L. Moutsianas, M. Mueller, N. Murugaesu, A. C. Need, P. O‘Donovan, C. A. Odhams, C. Patch, D. Perez-Gil, M. B. Pereira, J. Pullinger, T. Rahim, A. Rendon, T. Rogers, K. Savage, K. Sawant, R. H. Scott, A. Siddiq, A. Sieghart, S. C. Smith, A. Sosinsky, A. Stuckey, M. Tanguy, A. L. Taylor Tavares, E. R. A. Thomas, S. R. Thompson, A. Tucci, M. J. Welland, E. Williams, K. Witkowska, S. M. Wood, Sam Behjati, Matthew J. Murray, Catherine E. Hook, Patrick Tarpey

**Affiliations:** 1 East-Genomics Laboratory Hub (GLH) Genetics Laboratory, Cambridge University Hospitals NHS Foundation Trust, Cambridge, CB2 0QQ UK; 2 Department of Clinical Genetics, Cambridge University Hospitals NHS Foundation Trust, Cambridge, CB2 0QQ UK; 3 Wellcome Trust Sanger Institute, Hinxton, Cambridge CB10 1SA UK; 4 Department of Histopathology, Cambridge University Hospitals NHS Foundation Trust, Cambridge, CB2 0QQ UK; 5 Department of Neuropathology, Cambridge University Hospitals NHS Foundation Trust, Cambridge, CB2 0QQ UK; 6 Developmental Biology and Cancer Department, University College London Great Ormond Street Institute of Child Health, London, UK; 7 Department of Histopathology, Great Ormond Street Hospital for Children NHS Foundation Trust, London, WC1N 3JH UK; 8 Department of Paediatric Haematology and Oncology, Cambridge University Hospitals NHS Foundation Trust, Cambridge, CB2 0QQ UK; 9 Department of Pathology, University of Cambridge, Tennis Court Road, Cambridge, CB2 1QP UK; 10 Genomics England, Dawson Hall, Charterhouse Square, Barbican, London, EC1M 6BQ UK

**Keywords:** Cancer genomics, Cancer genomics

## Abstract

**Background:**

Whole-genome sequencing (WGS) of cancers is becoming an accepted component of oncological care, and NHS England is currently rolling out WGS for all children with cancer. This approach was piloted during the 100,000 genomes (100 K) project. Here we share the experience of the East of England Genomic Medicine Centre (East-GMC), reporting the feasibility and clinical utility of centralised WGS for individual children locally.

**Methods:**

Non-consecutive children with solid tumours were recruited into the pilot 100 K project at our Genomic Medicine Centre. Variant catalogues were returned for local scrutiny and appraisal at dedicated genomic tumour advisory boards with an emphasis on a detailed exploration of potential clinical value.

**Results:**

Thirty-six children, representing one-sixth of the national 100 K cohort, were recruited through our Genomic Medicine Centre. The diagnoses encompassed 23 different solid tumour types and WGS provided clinical utility, beyond standard-of-care assays, by refining (2/36) or changing (4/36) diagnoses, providing prognostic information (8/36), defining pathogenic germline mutations (1/36) or revealing novel therapeutic opportunities (8/36).

**Conclusion:**

Our findings demonstrate the feasibility and clinical value of centralised WGS for children with cancer. WGS offered additional clinical value, especially in diagnostic terms. However, our experience highlights the need for local expertise in scrutinising and clinically interpreting centrally derived variant calls for individual children.

## Background

Following the completion of the ‘first draft’ of the human cancer genome in recent years, culminating in meta-analyses of several thousand cases, comprehensive genomic readouts are beginning to enter oncological practice [1]. Reports from different centres indicate that such data, obtained through a variety of assays, may provide clinically meaningful insights that aid diagnoses, guide treatment, and inform prognosis [2]. In most countries, clinical cancer genomics has evolved from, and been integrated into, academic research efforts. The National Health Service of England (NHSE), through its subsidiary Genomics England, has adopted a different approach and will provide the genetically most informative singular assay, whole-genome sequencing (WGS), independent of clinical and academic institutions. Following the NHS core principle of equitable care, NHSE and Genomics England aim to provide paired tumour/normal WGS to deliver somatic and germline variation within a clinically meaningful timeframe, for every child diagnosed with cancer, irrespective of where they are treated in England. This aspiration has the potential to supersede sequential standard-of-care testing regimes.

Two features distinguish this effort from comparable programmes elsewhere. Most centres combine different sequencing techniques to provide high coverage of potentially actionable variant loci, through targeted re-sequencing, sensitive calling of gene fusions (typically through mRNA sequencing), and readouts of individual bases to assess patterns of mutations (signatures) [3] and copy number (next-generation sequencing (NGS)). By contrast, the strategy of Genomics England is to provide, in the first instance, WGS exclusively. Furthermore, Genomics England does not deliver raw sequencing data directly to the molecular tumour boards responsible for management and treatment decisions. Instead, they partner with a commercial sequencing supplier (Illumina) to provide triaged variant calls, which are subsequently cascaded through Genomic Laboratory Hubs to individual molecular tumour boards for multidisciplinary team meeting (MDT) discussion. For example, our institution served as the regional Genome Medicine Centre (1 of 13 at the time of our study) for 3 paediatric haematology and oncology centres in the East of England that is served by 2 dedicated paediatric solid tumour boards.

The infrastructure for tissue acquisition, processing, central generation and analysis of data, and regional molecular tumour boards have been established through the now completed 100,000 Genomes Project (2012–2018) of NHS England. The aim of this project had been to generate WGS data for patients suffering from rare diseases or cancer and to pilot the integration of genomic data into clinical decision making. Here, we report the feasibility and clinical utility of childhood cancer WGS, as implemented by Genomics England during the pilot phase, based on the experience of our regional East of England Genomic Medicine Service.

## Methods

### Recruitment and consent

Patients were recruited by a dedicated research nurse who ensured the mandatory provision of written parental consent prior to attainment of suitable fresh frozen tumour tissue and paired normal blood. The potential of WGS to return germline variants was discussed at the time of consent and a consultant clinical geneticist was present at the Genomic Tumour Advisory Board (GTAB) meeting to action appropriate follow-up including genetic counselling. Inclusion criteria were all children, aged 0–16 years at presentation, with a solid tumour diagnosis, availability of adequate fresh frozen tissue and germline DNA, and appropriate consent. There were no exclusion criteria.

### Sequencing

DNA from the paired tumour (fresh-frozen tissue) and matched normal (blood) samples were prepared locally using established diagnostic protocols. DNA sequencing was performed centrally at the NHS Genomics Medicine Sequencing Centre in Hinxton, Cambridge, UK. Sequencing library preparation was performed without polymerase chain reaction (PCR) unless the sample was limited, in which case an alternate PCR protocol was pursued (nano-prep). Sequencing was performed to a mean coverage of approximately 100X in the tumour and 40X in the paired normal sample.

### Data analysis

Somatic variant calling was performed using a suite of established variant calling algorithms to deliver substitutions and indels (SNV), copy number aberrations (CNA) and structural variants (SV) [4–6]. Reports were returned as annotated HTML files with high-quality SNV variants triaged into ‘Domains’ based on clinical actionability (Domain 1), non-actionable cancer census genes (Domain 2), or non-cancer genes (Domain 3) [7]. Global patterns of mutation were annotated for tumour mutation burden and COSMIC mutational signatures [3]. Germline variant delivery focused on non-synonymous SNVs in specific genes pertinent to each cancer type as defined by PanelApp [8] (see Supplementary Tables 1 and 3). Genome-wide CNA was depicted linearly via commercial software (BaseSpace Variant Interpreter-Illumina, Inc.) and overall mutational profiles informed via Circos plots (Supplementary Fig. 1).

### Clinical review

A summary of the clinically pertinent events was reviewed at the weekly tertiary Cambridge paediatric oncology GTAB meeting with mandatory clinical representation from paediatric oncology, pathology and clinical genetics. A critical component of the GTAB discussion was a formal evaluation of how the addition of WGS informed diagnosis, prognosis, or therapeutic opportunity. This appraisal is built on the wide multidisciplinary expertise and potential clinical trial opportunities. Genome data were reviewed in the context of the patient’s medical status and cancer family history, and clinical evaluation was formally documented as an MDT outcome and via mandatory questionnaire submission to NHSE using pre-defined impact options.

Variants predicted to change clinical management were confirmed via orthologous assay prior to formal reporting. Where indicated, variant calls were scrutinised using bespoke tools by local genomic scientists.

## Results

### Study cohort and samples

**Fig. 1:**
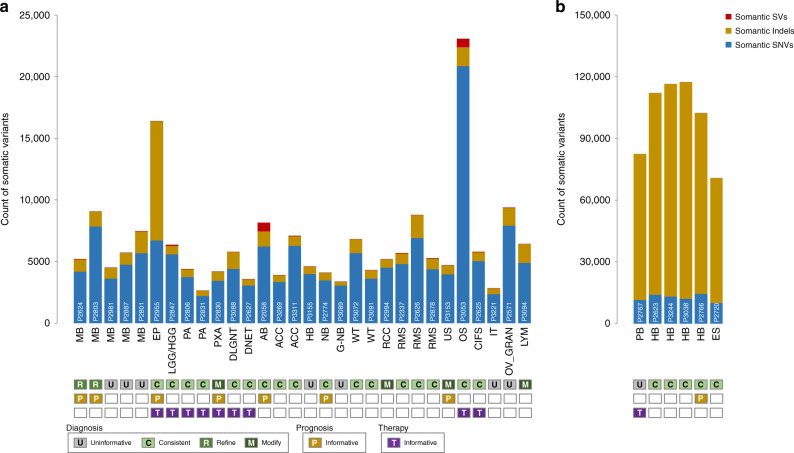
Bar chart indicating total variant counts in 36 childhood cancer genomes, and the clinical insight informed by these data. The panels beneath the bar chart indicate the clinical insight to inform diagnosis (U: uninformative, C: consistent, R: refine, M: modify), prognosis (P: informative) and therapy (T: informative) in each case. The number of somatically acquired indels was greatly elevated in samples prepared using a PCR based (nano-prep) protocol (**b**) compared to sequencing libraries prepared without PCR (**a**). MB medulloblastoma, EP anaplastic ependymoma, PB pineoblastoma, LGG/HGG biphasic neuroepithelial tumour, PA pilocytic astrocytoma, PXA glioma with molecular features of pleomorphic xanthoastrocytoma, DLGNT diffuse leptomeningeal glioneuronal tumour, DNET dysembryoplastic neuroepithelial tumour, AB astroblastoma, ACC adrenocortical carcinoma, HB hepatoblastoma, NB neuroblastoma, G-NB ganglio-neuroblastoma, WT Wilms’ tumour, RCC renal cell carcinoma, RMS rhabdomyosarcoma, US undifferentiated sarcoma, ES Ewing’s sarcoma, OS osteosarcoma, CIFS congenital infantile fibrosarcoma, IT immature teratoma, OV_GRAN ovarian granulosa cell tumour, LYM high-grade B cell lymphoma.

Non-consecutive NHS patients were recruited to the 100 K project by their treating clinician or oncology team within the East-GMC, led by Cambridge University Hospitals NHS Foundation Trust (CUH). Based on tissue availability and capacity of the 100 K project, tumour and germline DNA samples of 36 children (22 males, 14 females) were submitted for WGS (median age, 4 years, range 0.12–16.15 years) (Table 1, Fig. 1, and Supplementary Table 1). A varied range of 23 different tumour types was selected mainly comprising cancers of the central (CNS) and peripheral (PNS) nervous system (17 cases, 47%) and sarcoma (7 cases, 19%), among others. Prior standard-of-care analyses via fluorescence in situ hybridisation, immunohistochemistry, targeted NGS or single-gene assay (PCR and multiplex ligation-dependent probe amplification) was performed in 32 of the 36 (89%) cases (Supplementary Tables 1 and 4).

**Table 1:** Clinical detail of 36 cancer cases indicating the working diagnosis, and how this was influenced following whole-genome sequencing.

Sample	Sex	Age (years)	Cancer status	Tissue	Diagnosis	Revised/refined diagnosis
P2624	Female	2.19	Primary	CNS	Medulloblastoma (MB)—SHH-activated	MYCN-activated
P2803	Male	5.24	Primary	CNS	Medulloblastoma (MB)—classical, WNT-negative	SUFU-activated
P2981	Male	5.18	Primary	CNS	Medulloblastoma (MB)—non-WNT/non-SHH (group 4)	
P2887	Male	5.22	Primary	CNS	Medulloblastoma (MB)—non-WNT/non-SHH (group 4)	
P2801	Male	3.61	Primary	CNS	Medulloblastoma (MB)—myoblastic differentiation (group 3)	
P2955	Female	1.53	Primary	CNS	Anaplastic ependymoma (EP)	
P2767	Male	5.47	Primary	CNS	Pineoblastoma (PB)	
P2847	Male	10.77	Primary	CNS	Biphasic neuroepithelial tumour (LGG/HGG)	
P2806	Male	1.36	Recurrence	CNS	Pilocytic astrocytoma (PA)	
P2831	Male	3.81	Primary	CNS	Pilocytic astrocytoma (PA)	
P2830	Female	1.02	Primary	CNS	Glioma with molecular features of pleomorphic xanthoastrocytoma (PXA)	Infant-type hemispheric glioma
P3088	Male	9.46	Metastasis	CNS	Diffuse Leptomeningeal Glioneuronal Tumour (DLGNT)	
P2627	Male	7.63	Primary	CNS	Dysembryoplastic neuroepithelial tumour (DNET)	
P2058	Female	13.6	Primary	CNS	Astroblastoma (AB)	
P3269	Male	0.73	Primary	Adrenal	Adrenocortical carcinoma (ACC)	
P3311	Female	2.68	Primary	Adrenal	Adrenocortical carcinoma (ACC)	
P2623	Male	7.38	Primary	Liver	Hepatoblastoma (HB)	
P3244	Male	0.21	Recurrence	Liver	Hepatoblastoma (HB)	
P3038	Female	1.06	Primary	Liver	Hepatoblastoma (HB)	
P3155	Male	0.22	Primary	Liver	Hepatoblastoma (HB)	
P2766	Male	3.94	Metastasis	PNS	Neuroblastoma (NB)	
P2774	Male	0.89	Primary	PNS	Neuroblastoma (NB)	
P3089	Female	4.13	Primary	PNS	Ganglio-neuroblastoma (G-NB)	
P3072	Male	15.14	Metastasis	Renal	Wilms’ tumour (WT)	
P3091	Male	3.31	Primary	Renal	Wilms’ tumour (WT)	
P2994	Male	10.96	Primary	Renal	Renal cell carcinoma (RCC)	Wilms-like tumour
P2337	Female	1.28	Primary	Sarcoma	Rhabdomyosarcoma (RMS)	
P2626	Male	9.84	Primary	Sarcoma	Rhabdomyosarcoma (RMS)	
P2878	Female	1.68	Primary	Sarcoma	Rhabdomyosarcoma (RMS)	
P3153	Male	16.15	Primary	Sarcoma	Undifferentiated sarcoma (US)	BCOR-CCNB3 mutated sarcoma
P2720	Female	12.4	Primary	Sarcoma	Ewing’s sarcoma (ES)	
P3053	Female	13.6	Primary	Sarcoma	Osteosarcoma (OS)	
P2625	Female	0.12	Primary	Sarcoma	Congenital infantile fibrosarcoma (CIFS)	
P3221	Female	13.36	Primary	Teratoma	Immature teratoma (IT)	
P2571	Female	2	Primary	Ovarian	Ovarian granulosa cell tumour (OV_GRAN)	
P3094	Male	5.72	Primary	Lymphatic	High-grade B cell lymphoma (LYM)	MLLT10-DDX3X mutated lymphoma

### Overview of somatic variation

Total counts of somatic substitutions, small indels and structural variants were reported for each case (Fig. 1). In six tumours, a striking excess of indels correlated with a PCR library protocol (nano-prep) and were considered likely artefact (Fig. 1b). Other variant classes in these samples were unaffected, and the samples were retained for analysis. The remaining cases were relatively comparable across different cancer-types averaging 5200 substitutions, 1200 indels and 74 rearrangements. Outlier samples with a notable excess of genuine variants included P2955 (anaplastic ependymoma: 9666 indels), P2058 (astroblastoma: 721 SVs) and P3053: (osteosarcoma: 20,836 substitutions, 695 SVs) (Fig. 1).

**Fig. 2:**
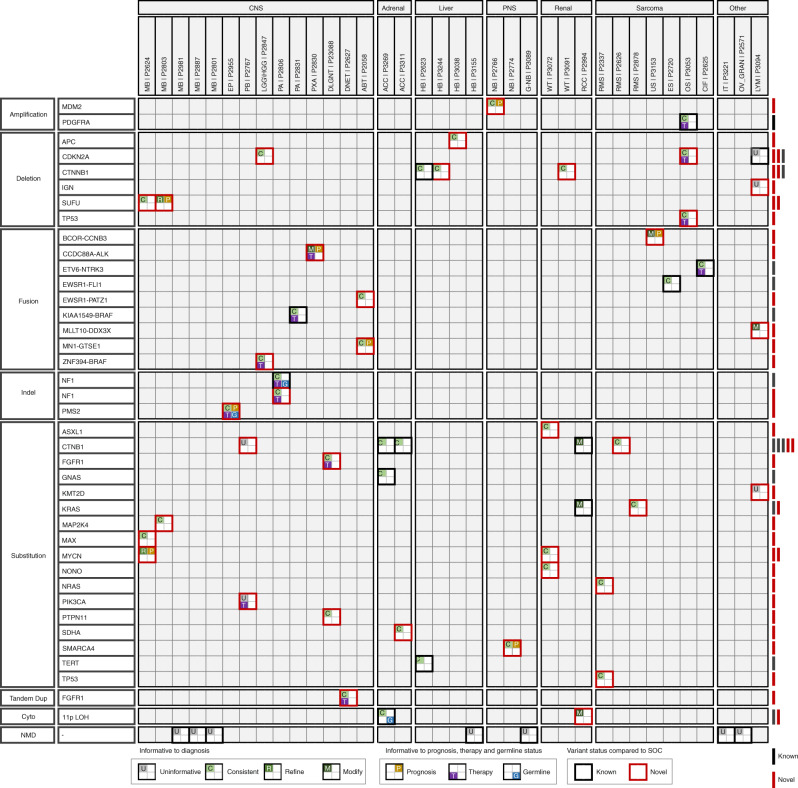
Detailed depiction of mutated cancer genes in 36 childhood cancer genomes, indicating the clinical outcome of each variant. For each variant, information on diagnosis (U: uninformative, C: consistent, R: refine, M: modify) prognosis (P: informative), therapy (T: informative) and germline status (G: informative) is indicated. Variants that were previously known via standard of care are indicated with black borders, whereas those which are novel are indicated with red borders. For each mutated gene, a tally of known and novel variants is presented as filled red or black bars on the right of each row.

A total of 52 variants were reported in the 36 samples (49 somatic, 3 germline). Driver somatic variants comprised 23 substitutions, 12 large deletions, nine gene fusions, two amplifications, one tandem duplication, one small indel and one case with reportable LOH. In seven tumours, no clearly deleterious germline or somatic variants were detected, despite extending the analyses from the default HTML to raw VCF files (Fig. 2). Of the reportable somatic variants, 12/49 (24%) were not present in the reported HTML files as they were either absent or had a ‘non-pass’ status, in the source VCFs (Supplementary Table 1). These cryptic variants included fusion genes involving *ALK* and *BRAF* and highlight the critical necessity to perform additional comprehensive local analyses of the raw data, particularly for imperfectly resolved cases. As genomes evolve into mainstream service, the ongoing development of variant detection and visualisation algorithms will substantially mitigate this risk.

### Impact on diagnosis

Of the 52 somatic and germline variants reported, 14 (27%) were previously known via standard-of-care management including germline uniparental disomy of chromosome 11p in a patient with a diagnosis of Beckwith-Wiedemann syndrome (P3269). The remaining 38/52 (73%) variants were novel and provided valuable additional insight into the working diagnosis in some patients.

The collective variants in each patient were either consistent with the working diagnosis (22/36, 61%), refined the diagnosis into a clinically distinct subtype (2/36, 6%), or changed the working diagnosis (4/36, 11%) (Fig. 1). Refined diagnoses were observed in two cases of medulloblastoma via the presence of a loss-of-function variant in *SUFU* (P2803) and an activating missense variant in *MYCN* (P2624). An example of a changed diagnosis includes the re-classification of a pleomorphic xanthoastrocytoma (P2830) to an infant-type hemispheric glioma [9], generated by an intrachromosomal deletion on chromosome 2p adjoining exons 1–12 of the *CCDC88A* gene with *ALK* exons 20–29 reported previously [10]. In seven cases (19%), no clearly deleterious germline or somatic variants were detected to influence available clinical status.

### Impact on the therapeutic opportunity

**Fig. 3:**
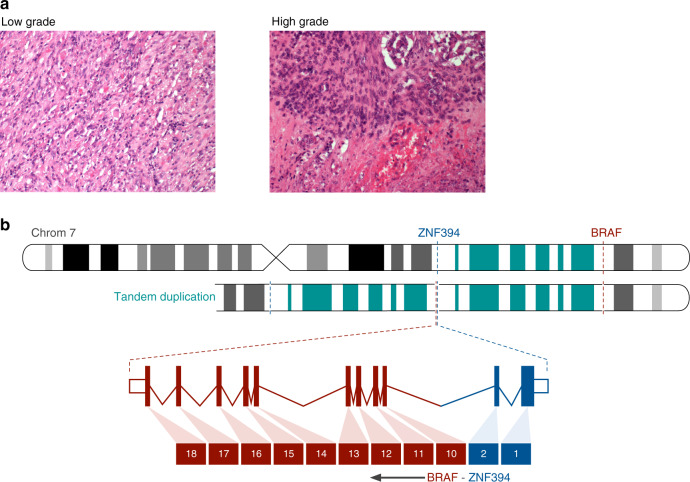
Novel *ZNF394-BRAF* fusion gene identified in patient P2847 via a tandem duplication on chromosome 7. The tumour specimen in this case was bi-phasic with both high- and low-grade components (**a**). The high-grade tissue was selected for whole-genome sequencing. The tandem duplication apposed exons 1–2 of *ZNF394* with exons 10–18 of the *BRAF* oncogene preserving the *BRAF* kinase domain consistent with functional validity (**b**).

We identified potential novel treatment opportunities in 8/36 patients (22%) via variants that would not have otherwise been routinely screened through current NHS practice. These included a missense mutation and an internal tandem duplication of the kinase domain in *FGFR1*, and structural variants leading to gene fusions in *BRAF* and *ALK* (Fig. 2). For example, in patient P2847, the identification of a novel gene fusion, (*ZNF394-BRAF*), led to the approval of MEK inhibitor therapy (Fig. 3).

### Impact on prognosis

Our findings informed the prognosis for 8 patients (22%). If the WGS data had been available at the time of diagnosis, the prognostic observations might have been influential to clinical management. Prognostically informative variants included amplification of the *MDM2* gene (P2766), associated with a poor prognosis in neuroblastoma [11], and a disease-defining *BCOR-CCNB3* fusion (patient P3153), which helped explain an unexpectedly long survival and relapse-free progression of a child originally diagnosed with an undifferentiated sarcoma carrying a poor prognosis [12]. A third case where prognostic mutations were particularly interesting (patient P2058) was a child with histologically-diagnosed astroblastoma but with uninformative global methylation profiling, considered to be the gold-standard diagnostic test for childhood brain tumours [13]. Here WGS revealed two putative driver fusions, *MN1-GTSE1* and *EWSR1-PATZ1*, described elsewhere in detail [14].

### Germline variation

Pathogenic germline variants were identified in three patients (8.3%), of which one (*PMS2*) was unknown via standard-of-care assays. The pathogenic indel in *PMS2* was identified in a patient with anaplastic ependymoma (P2955), confirmed on central pathology review. This patient presented with an elevated number of somatic indels consistent with congenital mismatch repair deficiency (CMMRD) (Fig. 1). During the project, bespoke diagnostic testing, which overcomes the complexity from the nearby pseudogene, *PMS2CL*, revealed an additional germline *PMS2* exon 12 deletion, confirming a diagnosis of CMMRD. A previously detected truncating germline mutation in *NF1* in a patient with pilocytic astrocytoma (P2806), was paired with a novel somatic *NF1* truncating indel and confirmed a clinical diagnosis of neurofibromatosis type 1 (NF1). The proportion of our cohort with pathogenic germline variants (8.3%) aligns closely with previous studies from large paediatric cohorts [15, 16].

### Added clinical insight of WGS

Detection of novel, clinically informative driver variants was concentrated in CNS/PNS tumours, with variants in 12/17 (70%) of these cases, adding additional clinical insight. In contrast, of the remaining 19 patients in our pilot cohort, novel clinical insight was constrained to four patients (21%), a lymphoma (P3094), an undifferentiated sarcoma (P3153), a renal cell carcinoma (P2994) and an osteosarcoma (P3053). Over half of the informative novel variants were SV or CNA (9/15 variants), indicating the specific utility of agnostic WGS for variants that would otherwise require multiple sequential targeted assays to uncover. This was exemplified in our patient with an *MN1* fusion-positive astroblastoma (P2058), where global methylation profiling was uninformative, and targeted *MN1* testing would have missed the second gene fusion detected, *EWSR1-PATZ1* [14].

## Discussion

Our data testify to the feasibility of integrating centralised WGS into routine NHS diagnostic practice and MDT working to deliver clinical benefits to individual children and their families.

The 100 K project did not grow organically from academic research initiatives, which typically commence with more targeted and limited molecular analyses. Instead, NHSE pursued a partnership with industry, using WGS as an unusual ‘entry point’ into the field. As a result, the project affords great opportunity for patients and their clinical teams, but also brings challenges that need to be considered and overcome as the service is being implemented nationally. Firstly, the analysis pipeline is unusual with central sequence generation and variant calling. Data is returned to regional genomics centres as variant files, for local, non-standardised interpretation of data. The practical implication of this observation is that the same centrally derived data set could be further analysed interpreted differently depending on local practice at individual sites. For example, bespoke interrogation of our data delivered additional critical variants in 10/36 cases. Currently, there are no formal mechanisms in place through which learning can be easily shared nationally. One way to overcome this would be to perform regular benchmarking exercises, i.e. identical centrally derived data sets to be analysed independently by local genomics services to systematically improve pipelines and clinical interpretation practice. This is of critical importance as the long-term aim of this project is not just to provide adjunct data, but to replace the current standard-of-care molecular techniques used in pathology practice. In this pilot study, turnaround times were protracted compared with alternate standard of care assays. However, our logistical and operational experience of the pilot phase has enabled a more rapid prospective clinical service, which can achieve turnaround times (from consent to report) as short as 1 month.

Previous reports of childhood cancer genomics programmes implemented elsewhere have stressed the novel therapeutic options that genomic readouts reveal [15, 16]. Our experience broadly corroborates this notion, with genomic analyses identifying potential new treatment options in 8/36 cases (22%). We would suggest, however, that the therapeutic utility may be limited outside clinical trials or children with an incurable disease. As attractive as non-cytotoxic agents may appear from an adverse effect profile, it would be questionable to deviate from established first-line treatment protocols, principally based on cytotoxic agents, that achieve high cure across most entities. For example, while *BRAF* fusions are common in low-grade glioma, first-line cytotoxic treatment with carboplatin-vincristine achieves long term survival in ~90% of children. Therefore, *BRAF* inhibitors are reserved for specific indications such as recurrent or refractory cases [17]. However, targeted therapies are under investigation in low-grade gliomas, such as an upcoming European trial of frontline MEK inhibition or studies of *BRAF* inhibition in V600E mutated tumours [18]. Each paediatric cancer specialist group needs to develop updated guidelines where the role, timing and duration of such novel agents is clarified [17, 19]. Our experience shows that perhaps the most useful aspect of WGS has been in refining or changing diagnoses; many childhood cancers have complex pathological nuances that DNA readouts help to address [14].

In absolute terms, our pilot cohort was relatively small. However, it represented over one-sixth of all paediatric cases recruited into the 100 K pilot by 13 centres nationally. Clearly, our experience with the 100 K project could be unique to our centre, which may be at an advantage in the interpretation of centrally derived data, given our institutional and personal track record of generating, analysing, and publishing genomic data, within the context of local academic institutions (University of Cambridge, Wellcome Sanger Institute). We will continue to report and publish our experience as the national programme goes ‘live’.

In summary, our analysis of 100 K project cases has demonstrated the clinical utility of integrating WGS into routine NHS testing for paediatric cancer. Our comprehensive MDT approach and experiences during data analysis, interpretation and clinical review have helped shape our future service, showing how it can fit in with current NHS practice. Routine WGS is set to make an important addition to the diagnosis and management of cancers, providing a detailed account of the DNA changes underpinning the individual tumours of our patients. Overcoming some of the highlighted challenges to nationwide implementation should ensure that this opportunity results in improved clinical outcomes for these patients, with the delivery of effective personalised medicine.

## Supplementary information

Supplementary Material Legends

Supplementary Figure 1

Supplementary Table 1

Supplementary Table 2

Supplementary Table 3

Supplementary Table 4

## Data Availability

Data are available upon reasonable request.
