# Supplementary material for: The NHS England 100,000 Genomes Project: feasibility and utility of centralised genome sequencing for children with cancer
Source: Br J Cancer. 2022 Apr 22;127(1):137–44. doi: 10.1038/s41416-022-01788-5 (PMC9276782; doi:10.1038/s41416-022-01788-5)
Supplement: Supplementary file 1 — Supplementary Material Legends [file 41416_2022_1788_MOESM1_ESM.docx]

**Supplementary Legends**

Supplementary Table 1

Clinical summary of 36 childhood cancer cases, sequencing performance metrics, prior standard of care (SOC) assays and clinical impact of the sequence variants identified in each case.

Supplementary Table 2

Somatic and germline variants identified in 36 childhood tumours, and clinical relevance of each mutation.

Supplementary Table 3

Germline genes interrogated in 36 childhood cancer cases. TYA: Teenager and young adult.

Supplementary Table 4

Prior standard of care (SOC) assays performed in 36 childhood cancer cases.

Supplementary Figure 1

CIRCOS and linear CNV plots derived from whole genome sequencing of 36 childhood cancers.
