# Supplementary material for: The NHS England 100,000 Genomes Project: feasibility and utility of centralised genome sequencing for children with cancer
Source: Br J Cancer. 2022 Apr 22;127(1):137–44. doi: 10.1038/s41416-022-01788-5 (PMC9276782; doi:10.1038/s41416-022-01788-5)

P2624 | Medulloblastoma (MB)

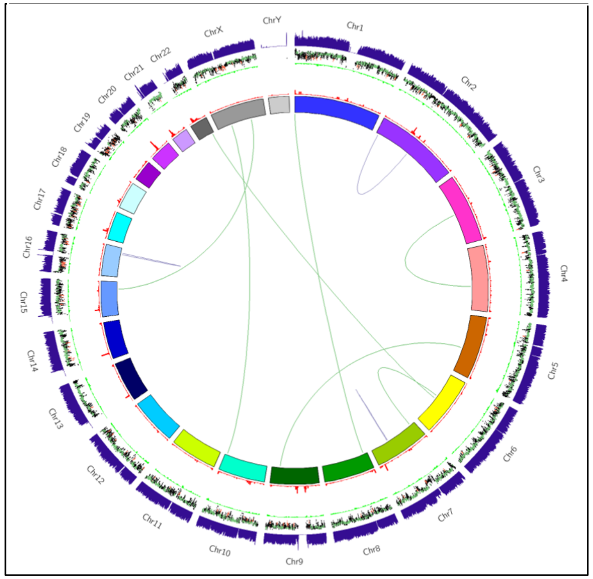

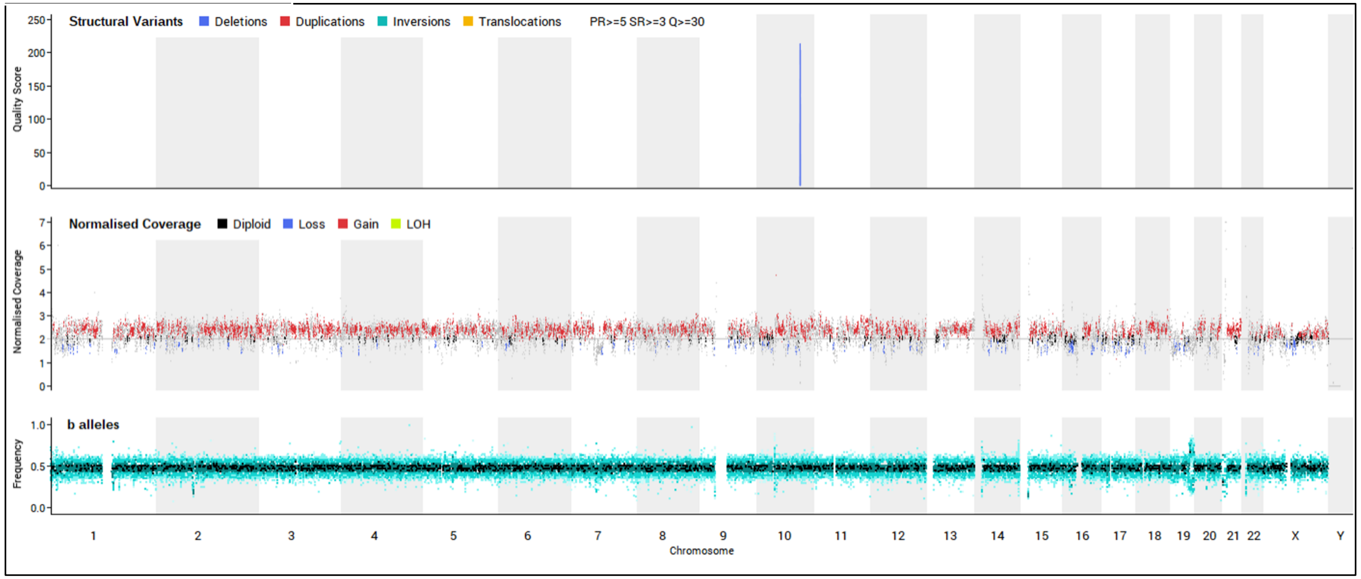

P2803 | Medulloblastoma (MB)

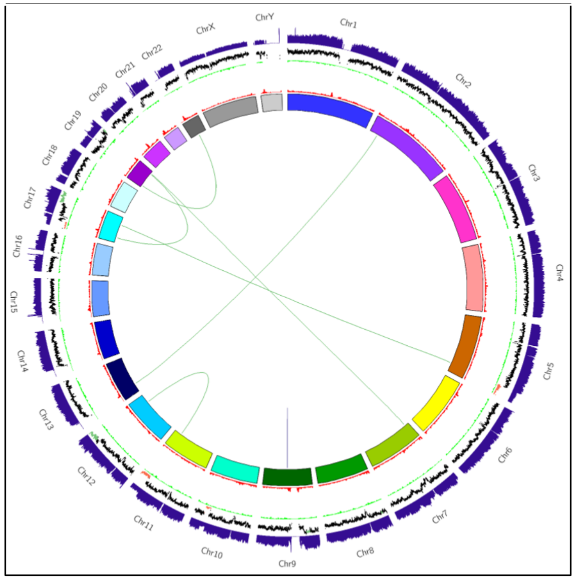

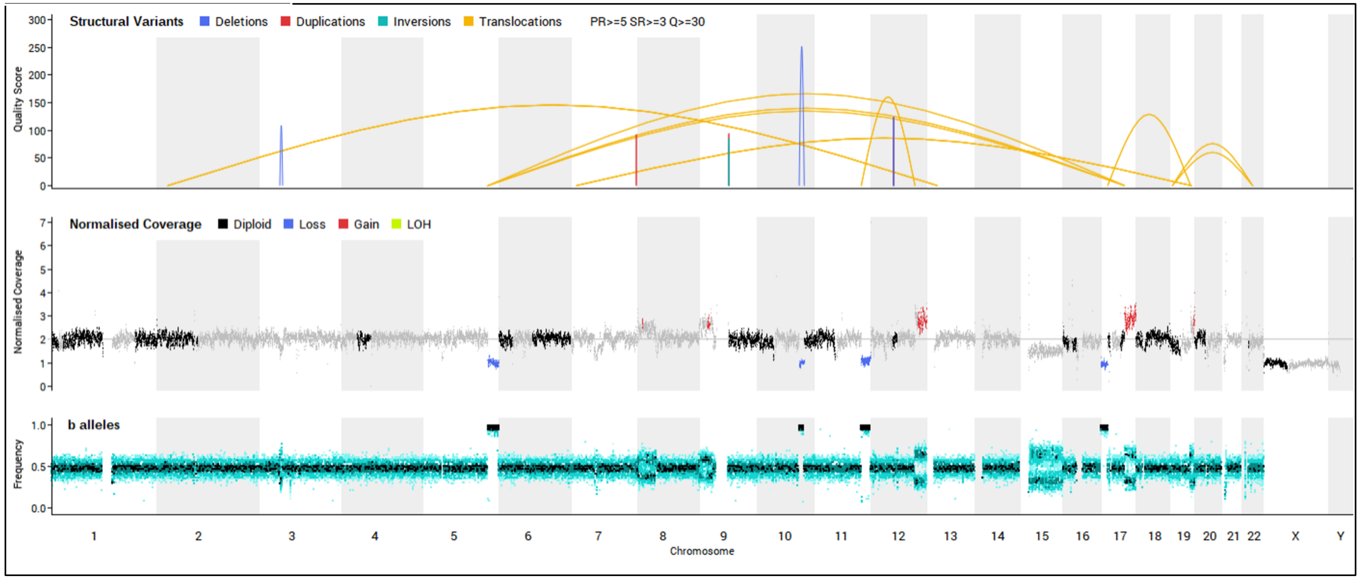

P2981 | Medulloblastoma (MB)

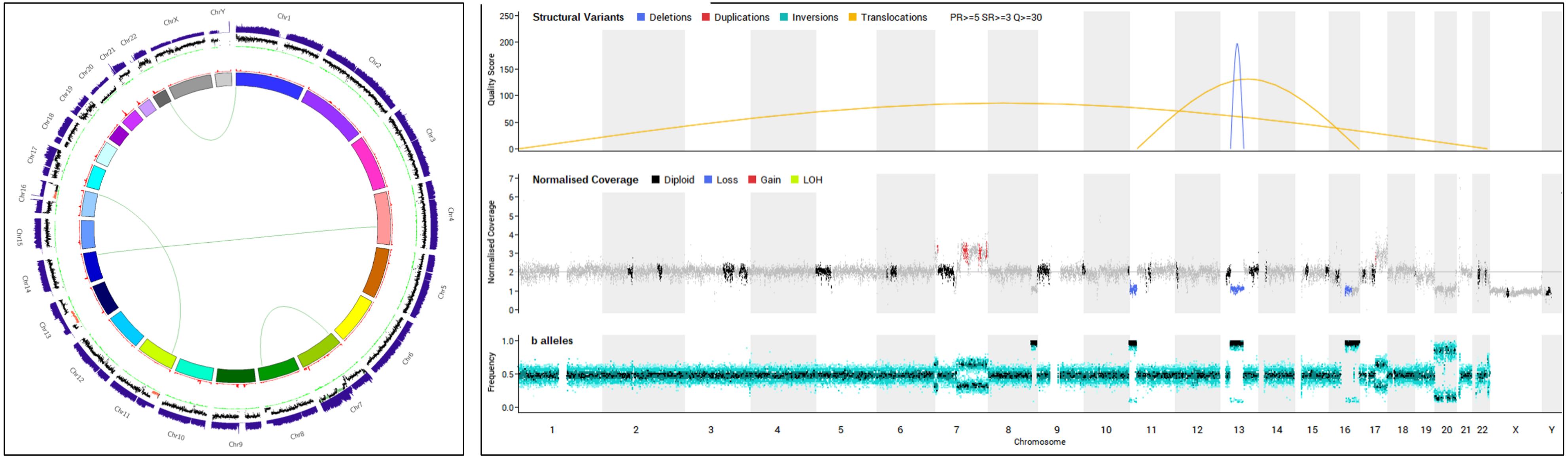

P2887 | Medulloblastoma (MB)

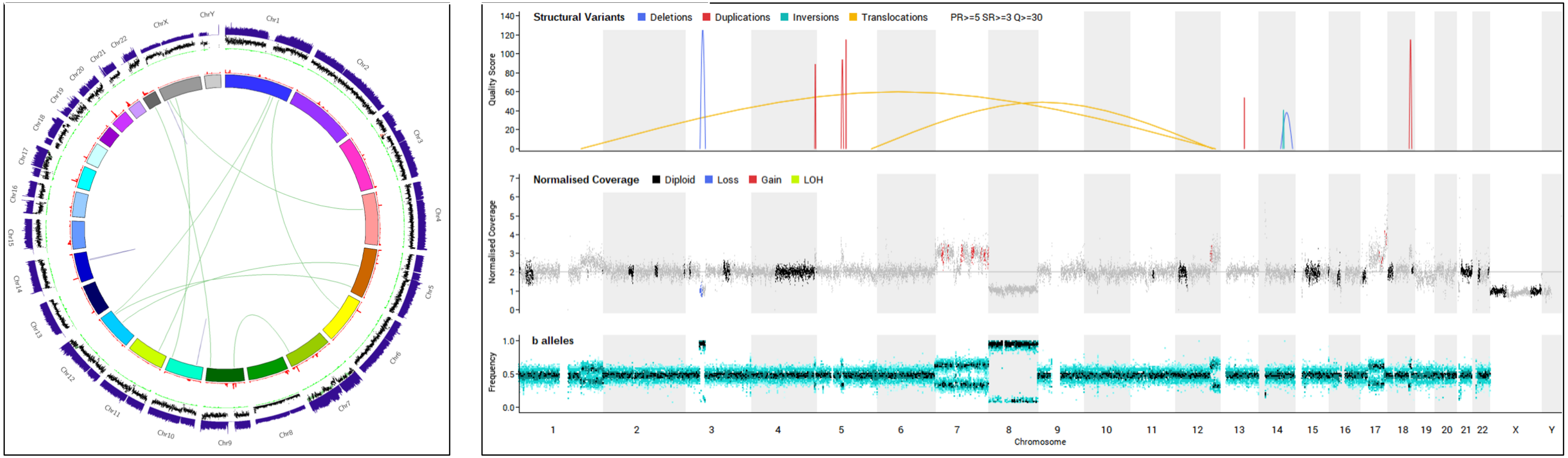

P2801 | Medulloblastoma (MB)

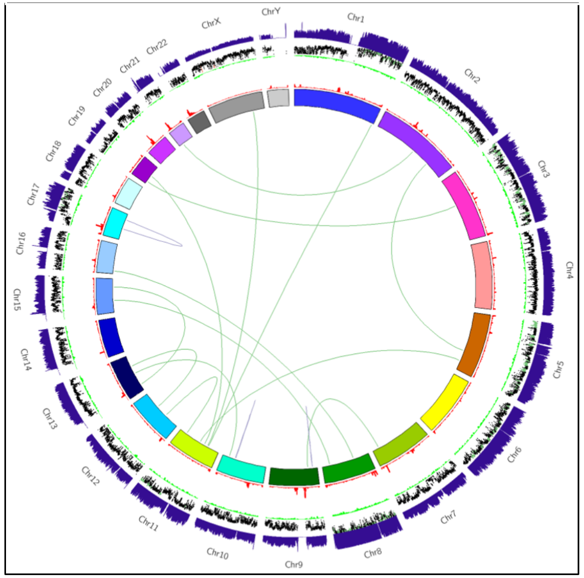

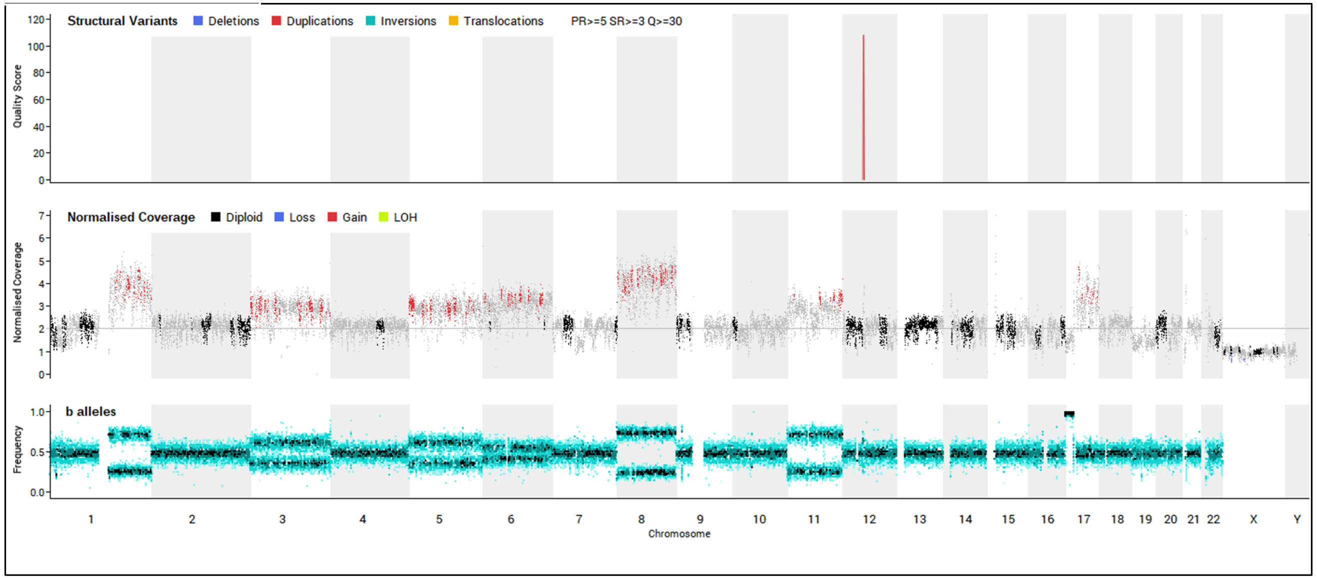

P2955 | Anaplastic ependymoma (EP)

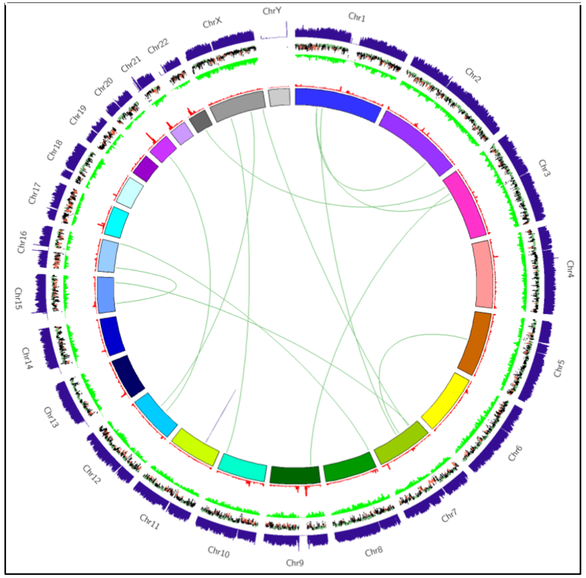

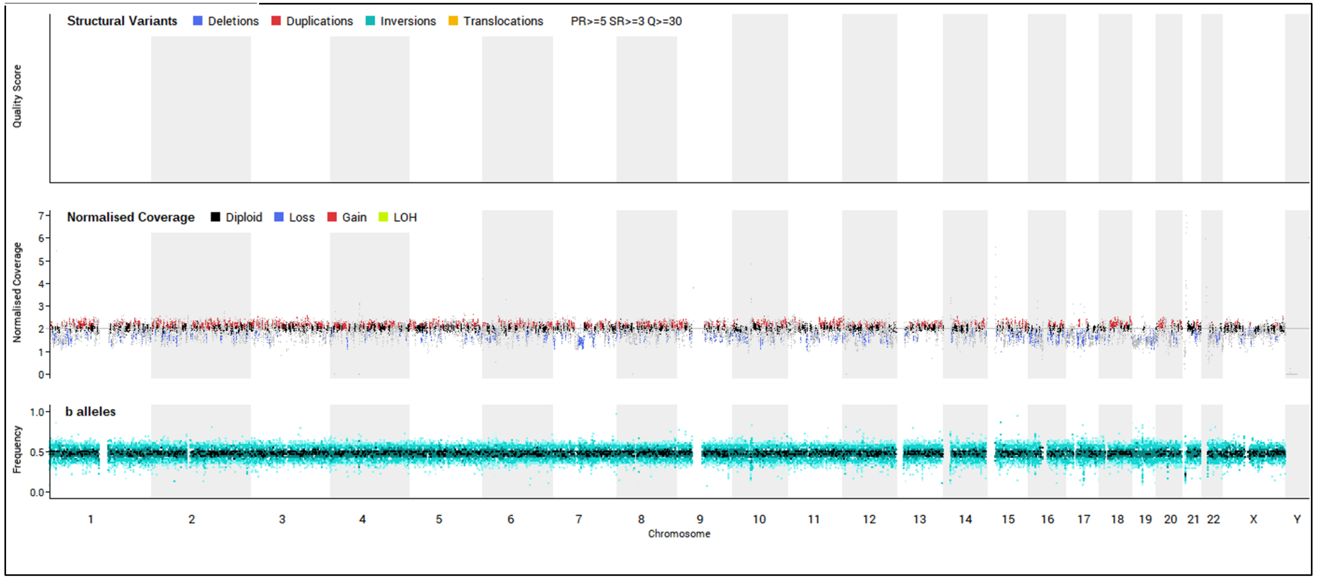

P2767 | Pineoblastoma (PB)

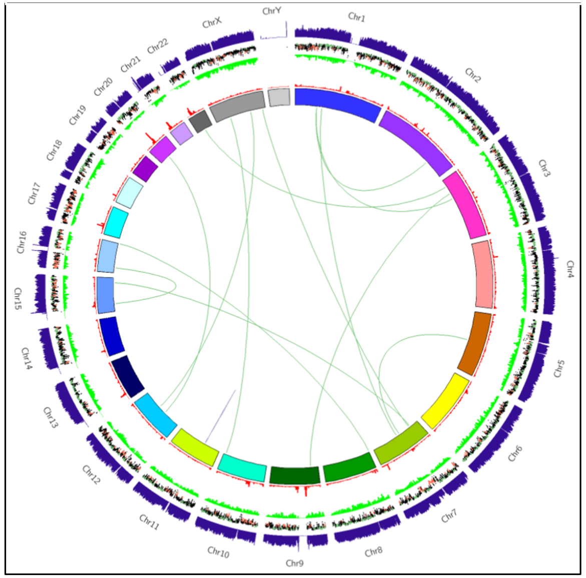

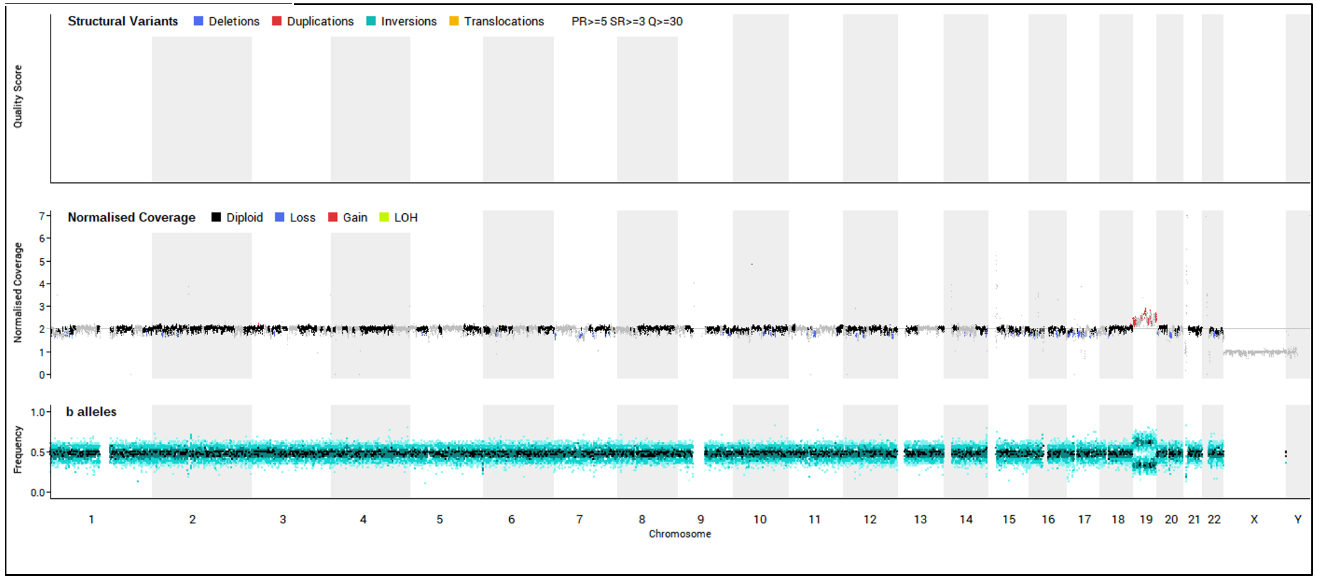

P2847 | Biphasic neuroepithelial tumour (LGG/HGG)

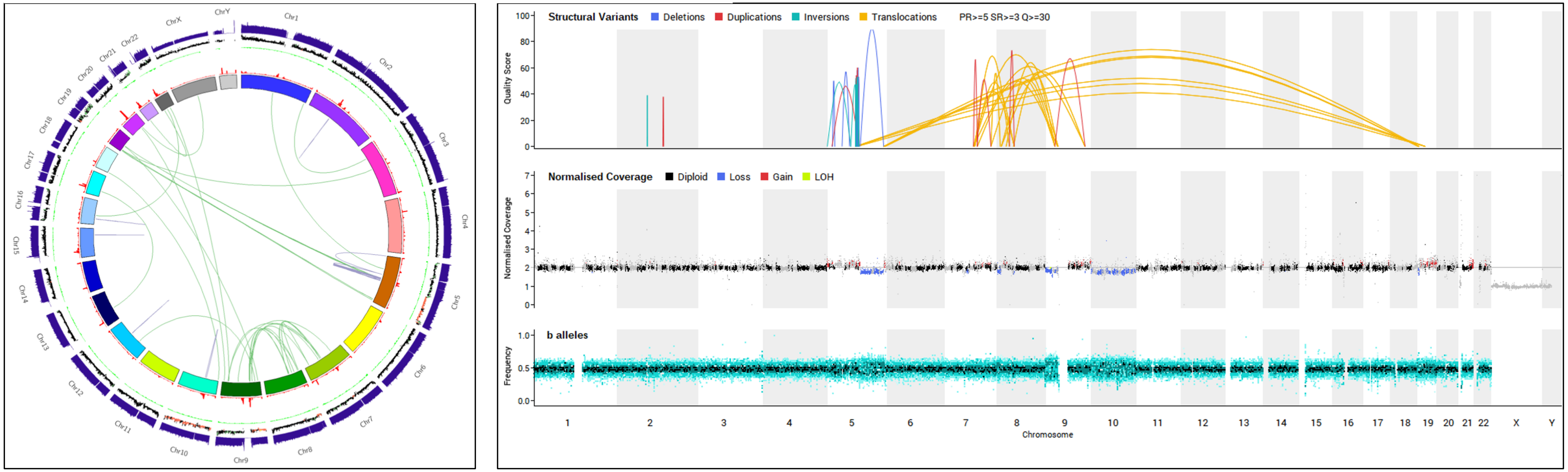

P2806 | Pilocytic astrocytoma (PA)

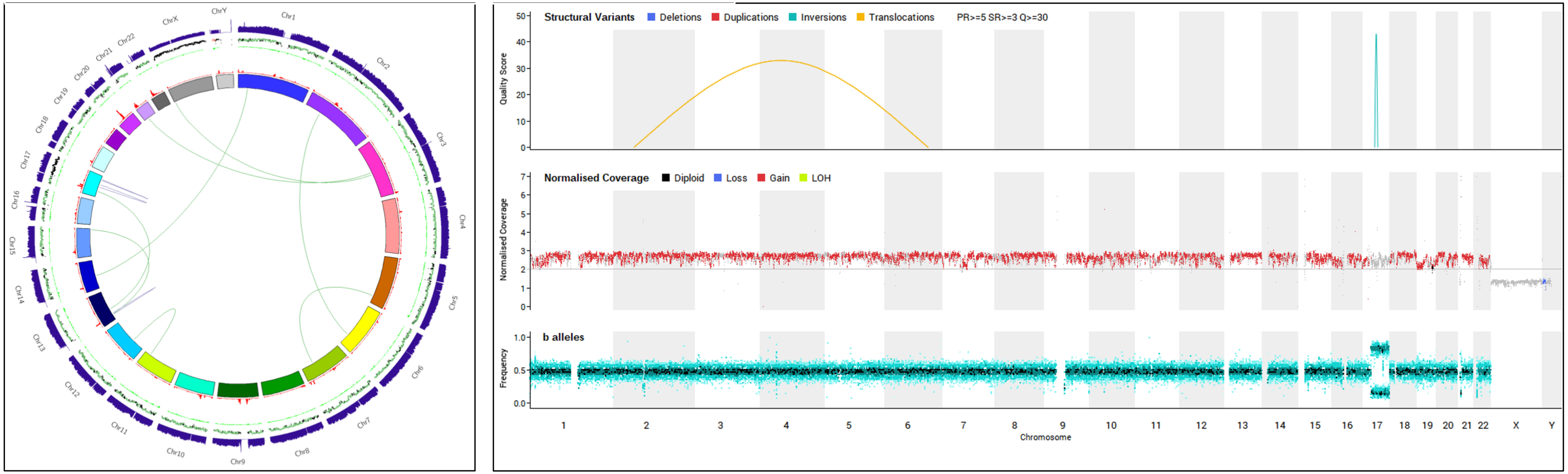

P2831 | Pilocytic astrocytoma (PA)

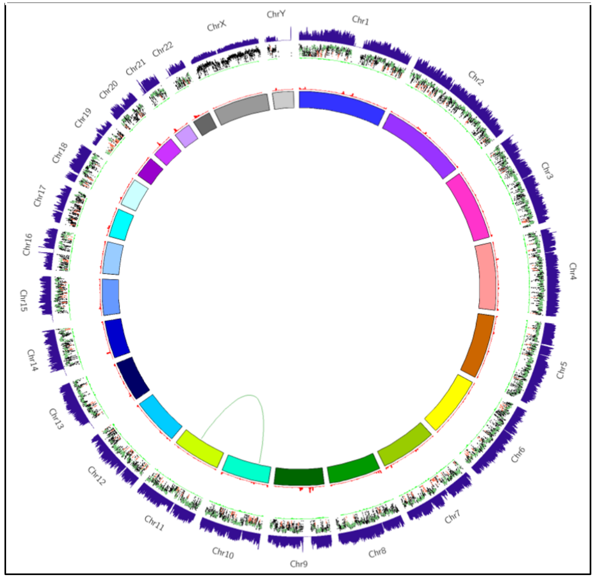

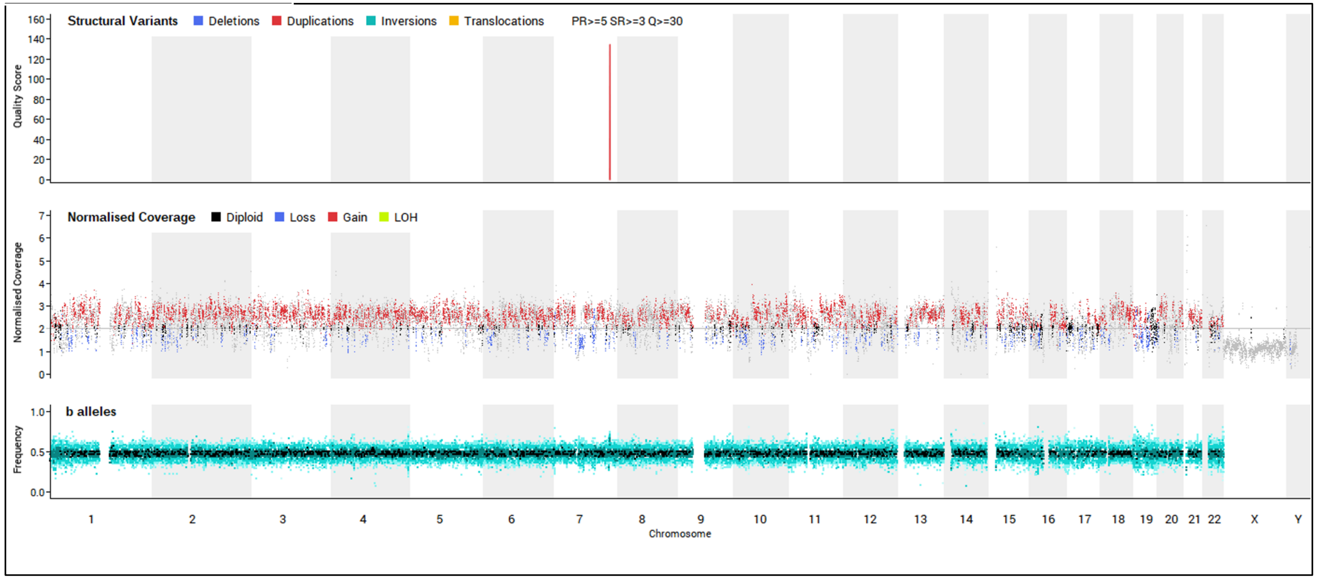

P2830 | Glioma with molecular features of pleomorphic xanthoastrocytoma (PXA)

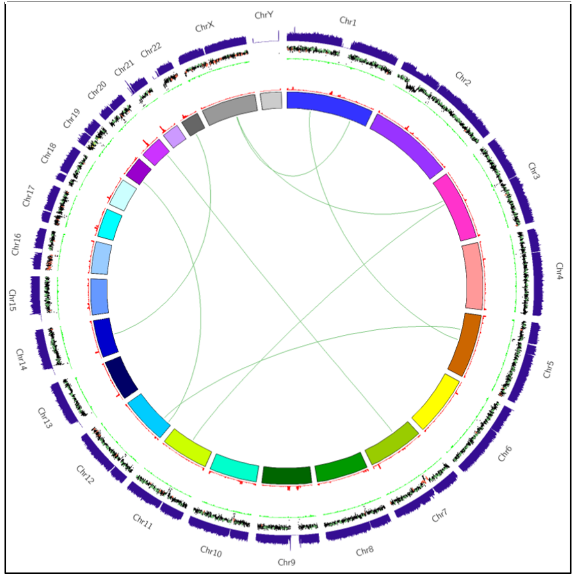

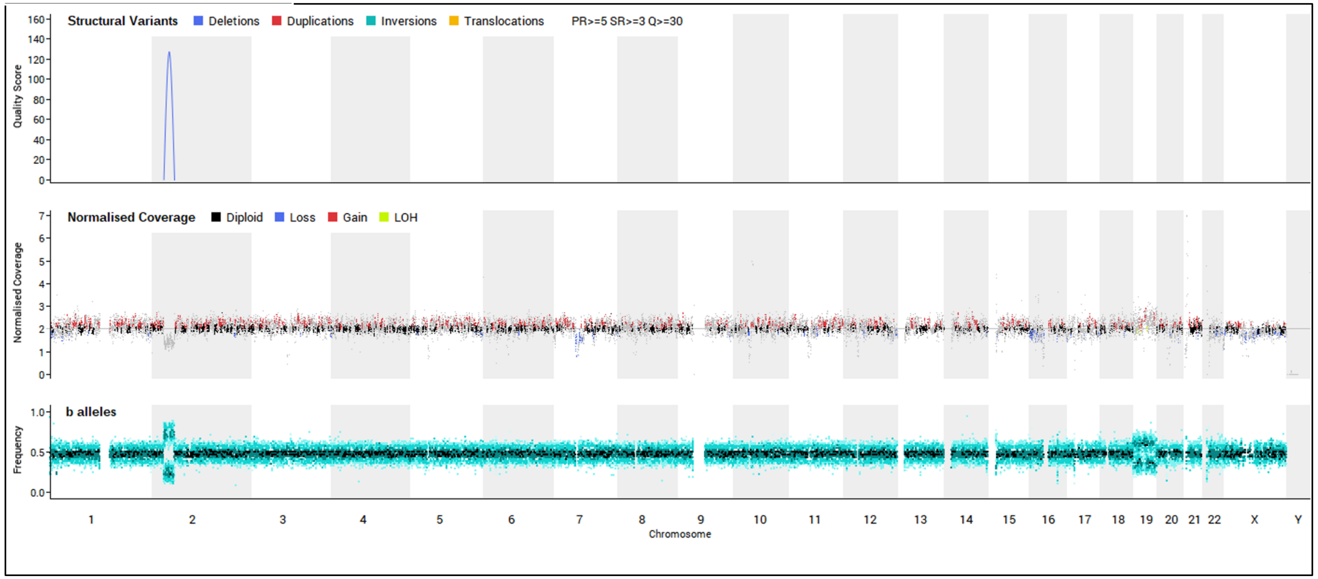

P3088 | Diffuse Leptomeningeal Glioneuronal Tumour (DLGNT)

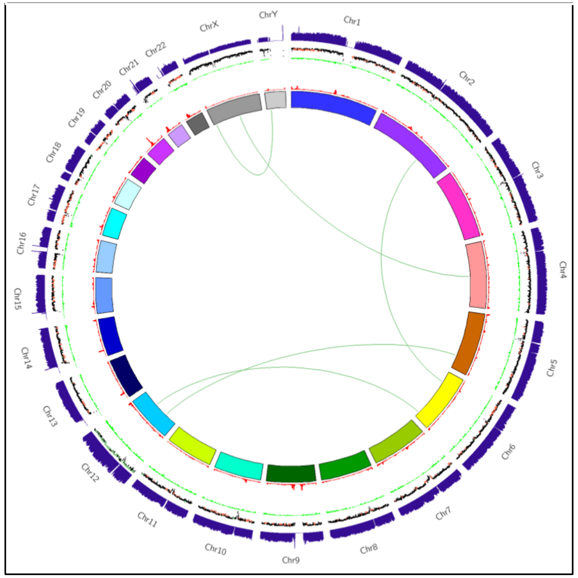

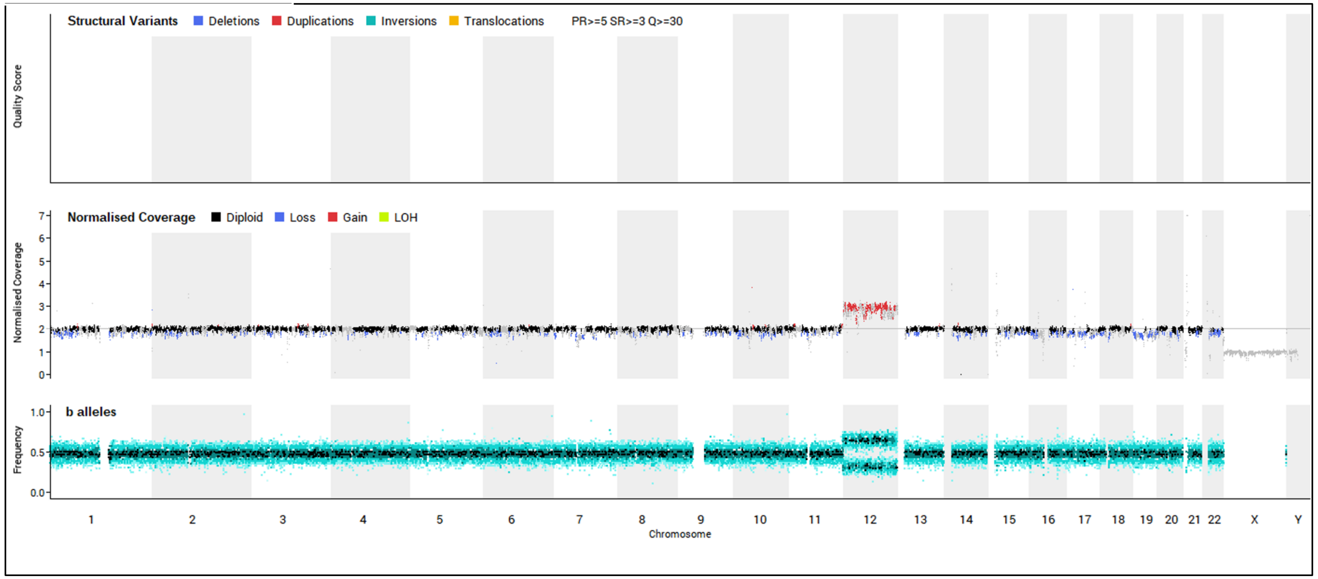

P2627 | Dysembryoplastic neuroepithelial tumour (DNET)

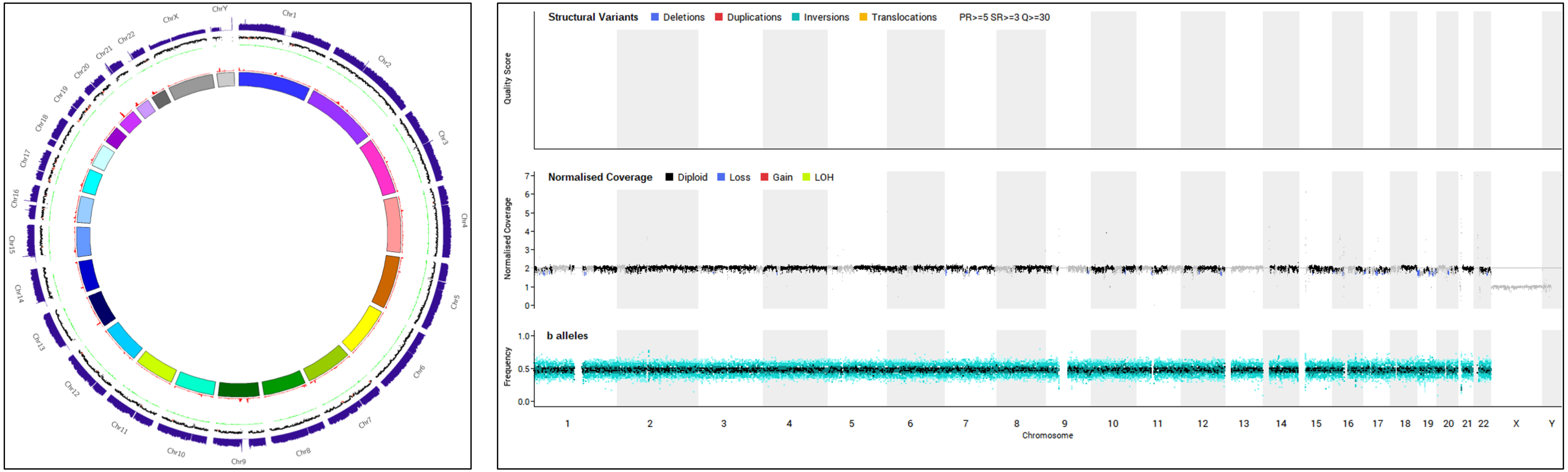

P2058 | Astroblastoma (AB)

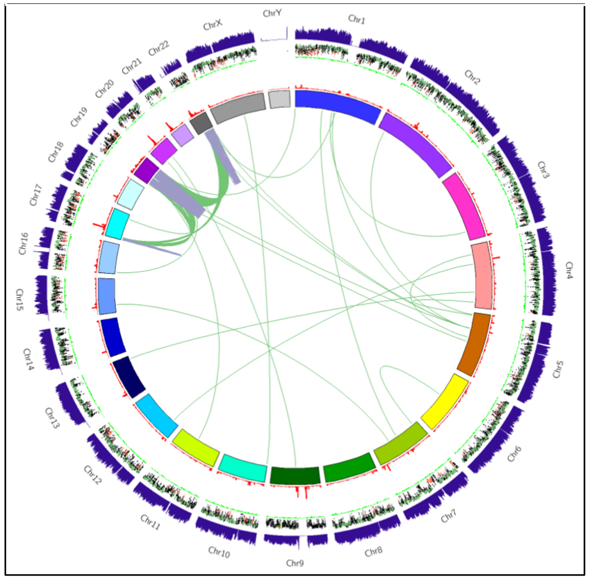

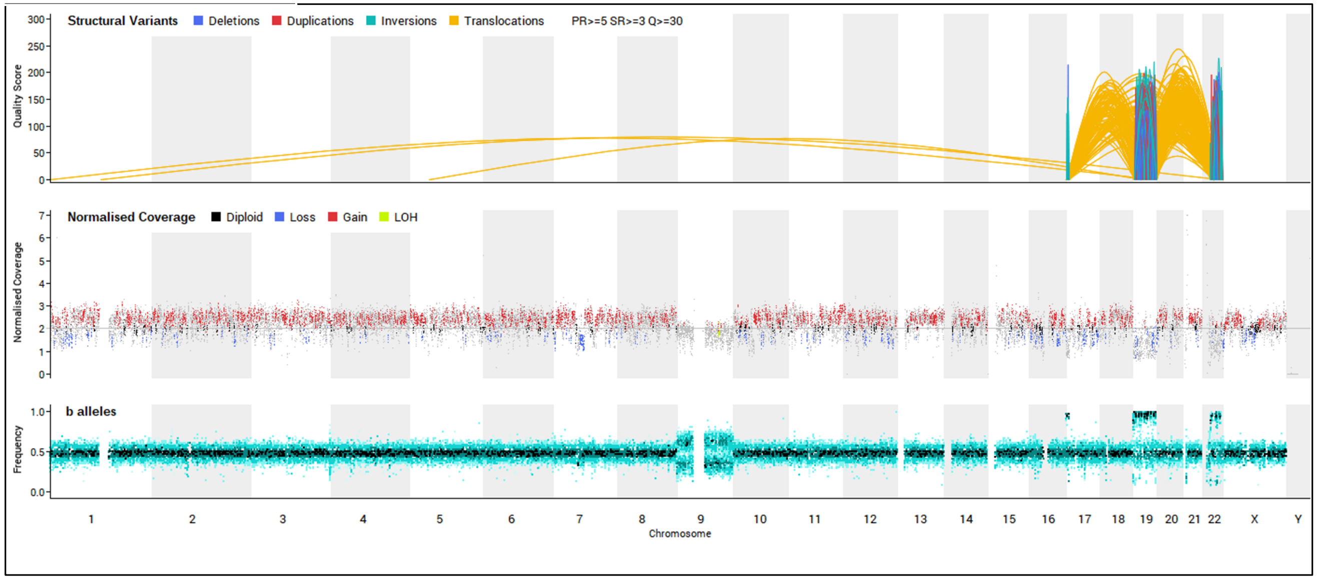

P3269 | Adrenocortical carcinoma (ACC)

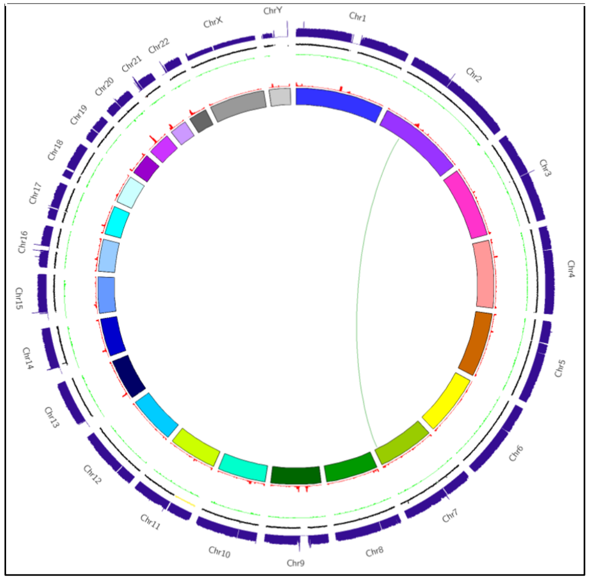

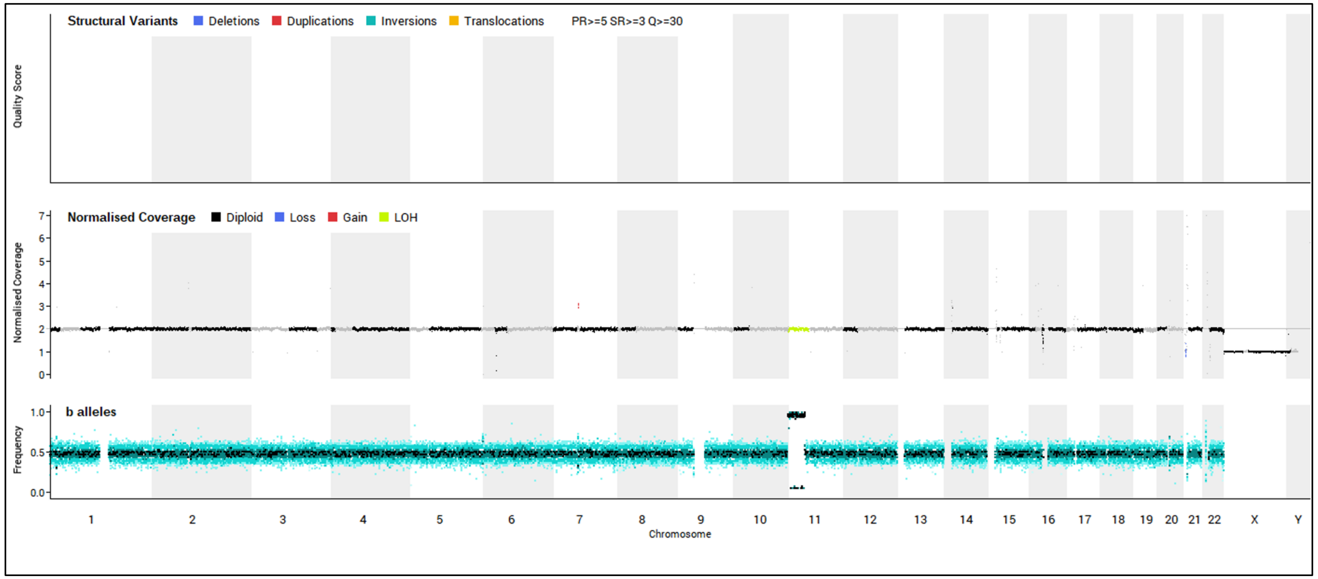

P3311 | Adrenocortical carcinoma (ACC)

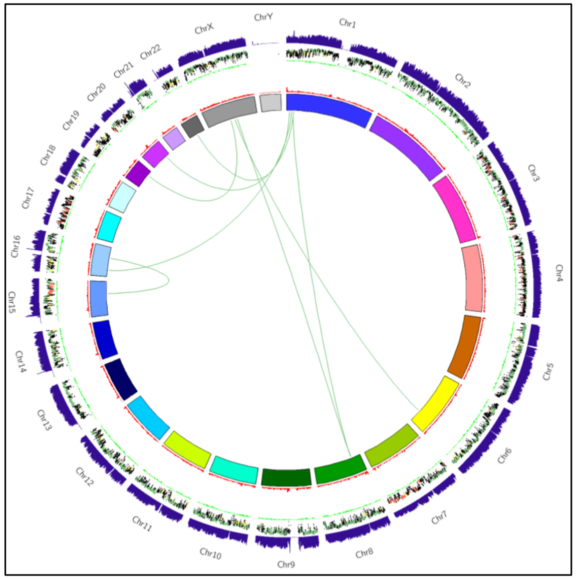

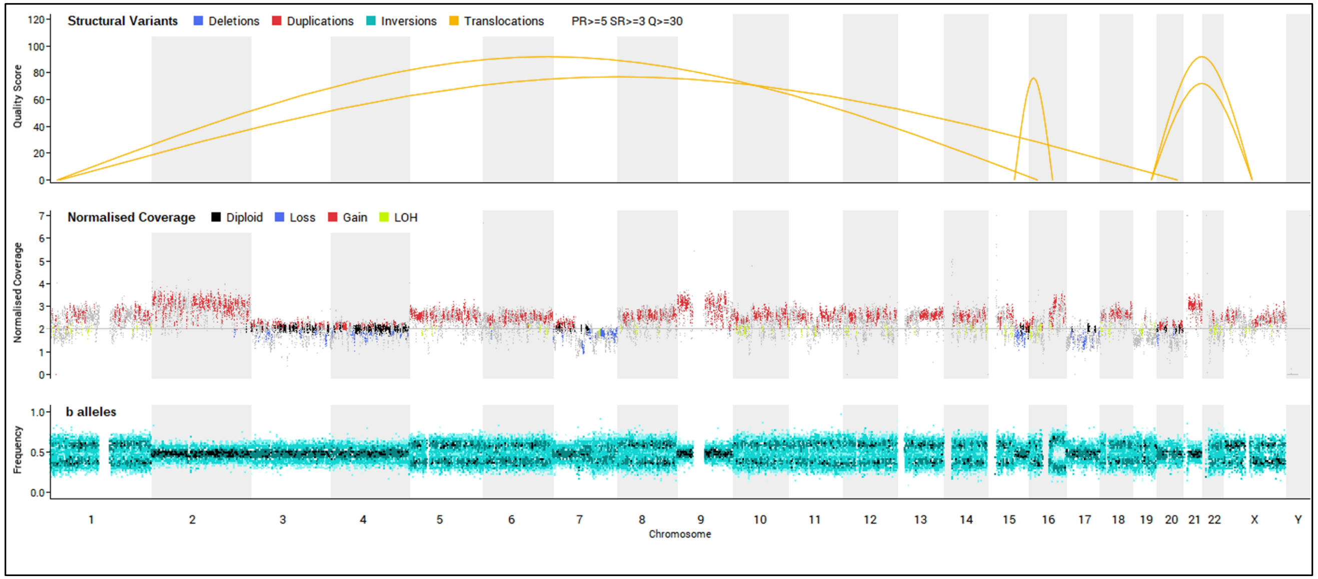

P2623 | Hepatoblastoma (HB)

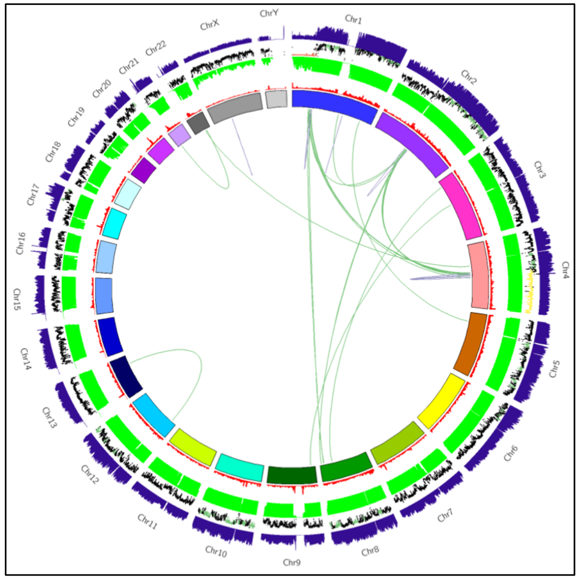

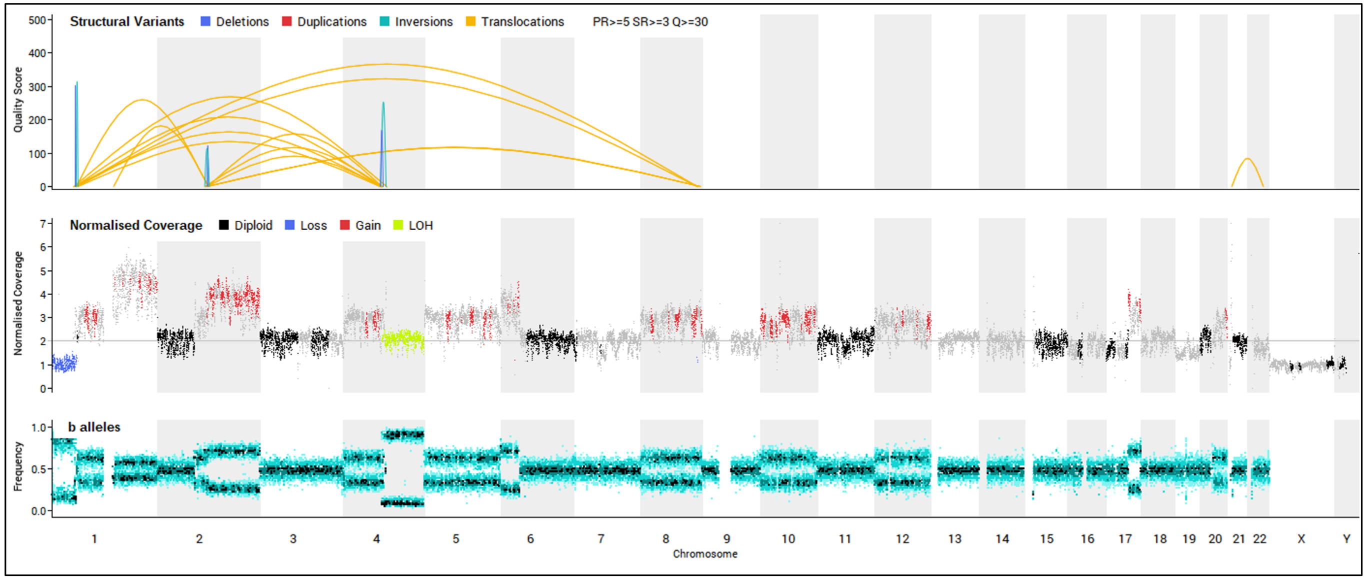

P3244 | Hepatoblastoma (HB)

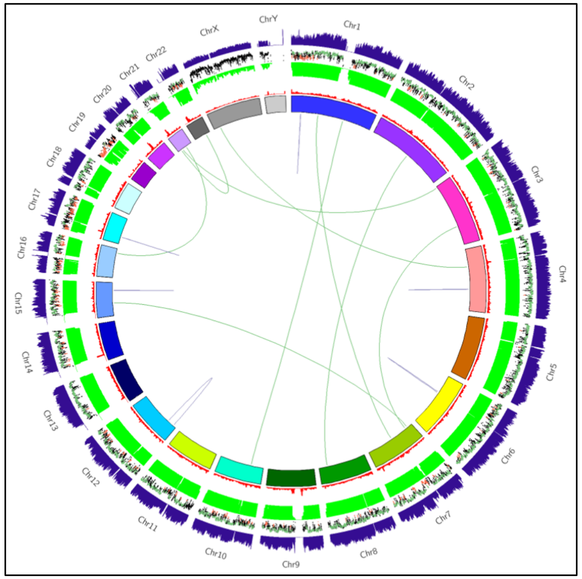

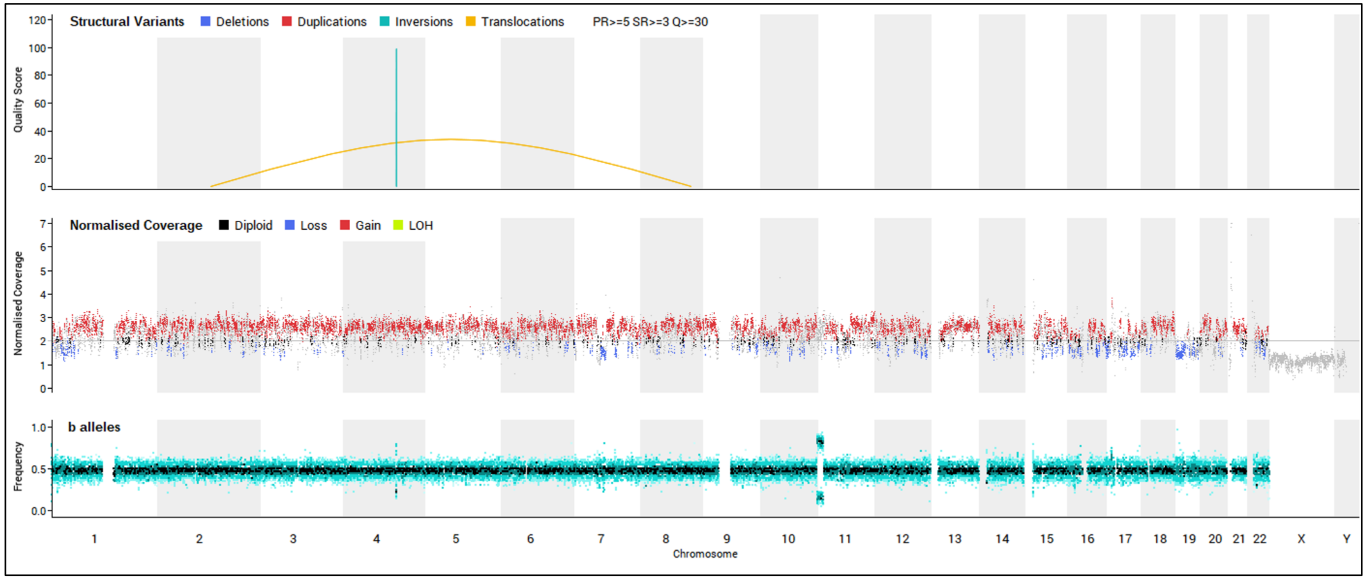

P3038 | Hepatoblastoma (HB)

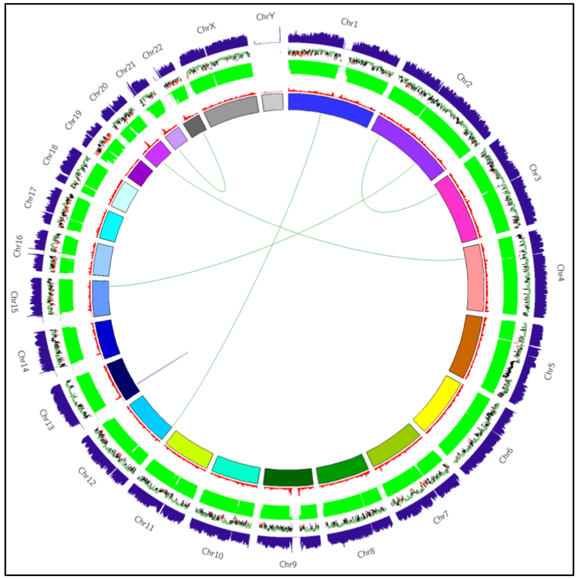

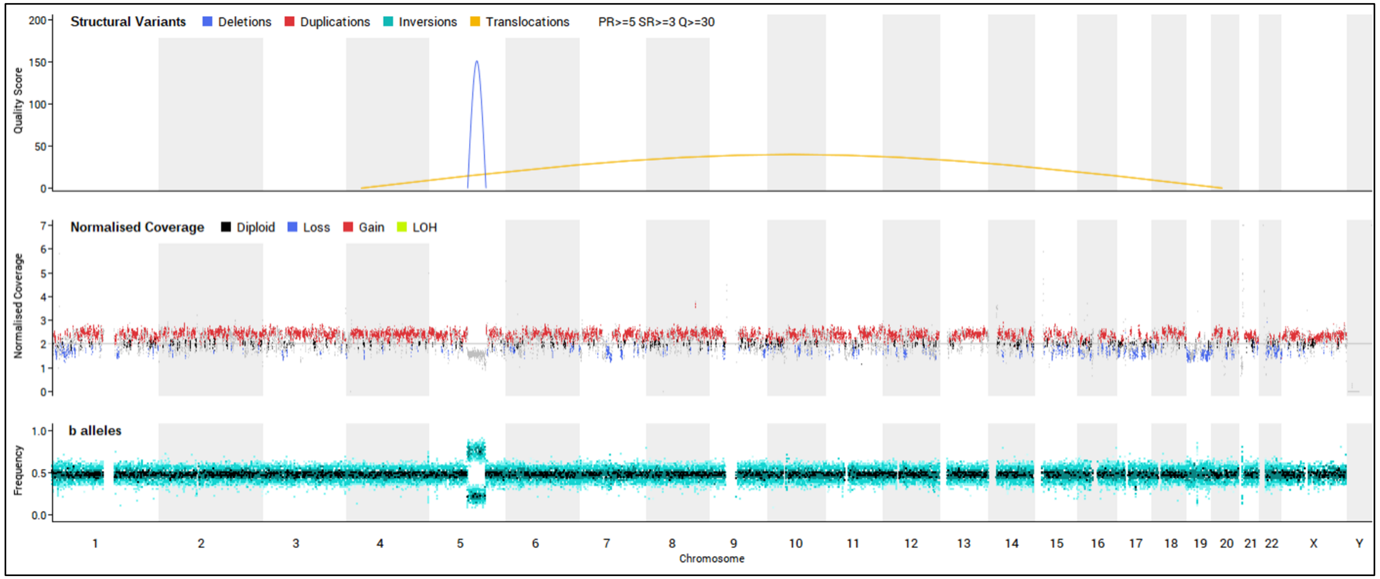

P3155 | Hepatoblastoma (HB)

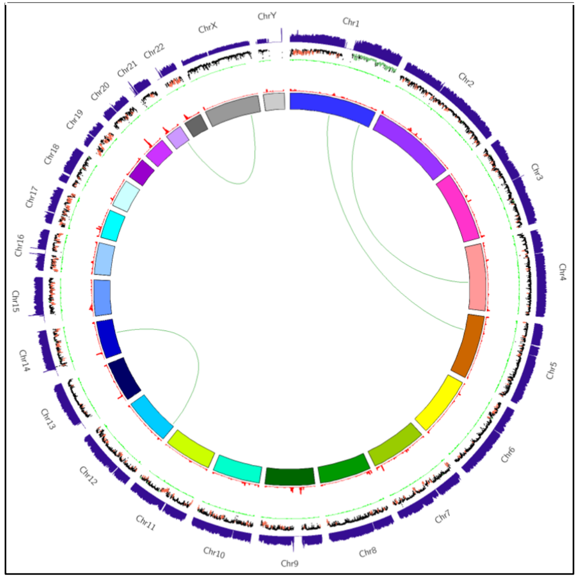

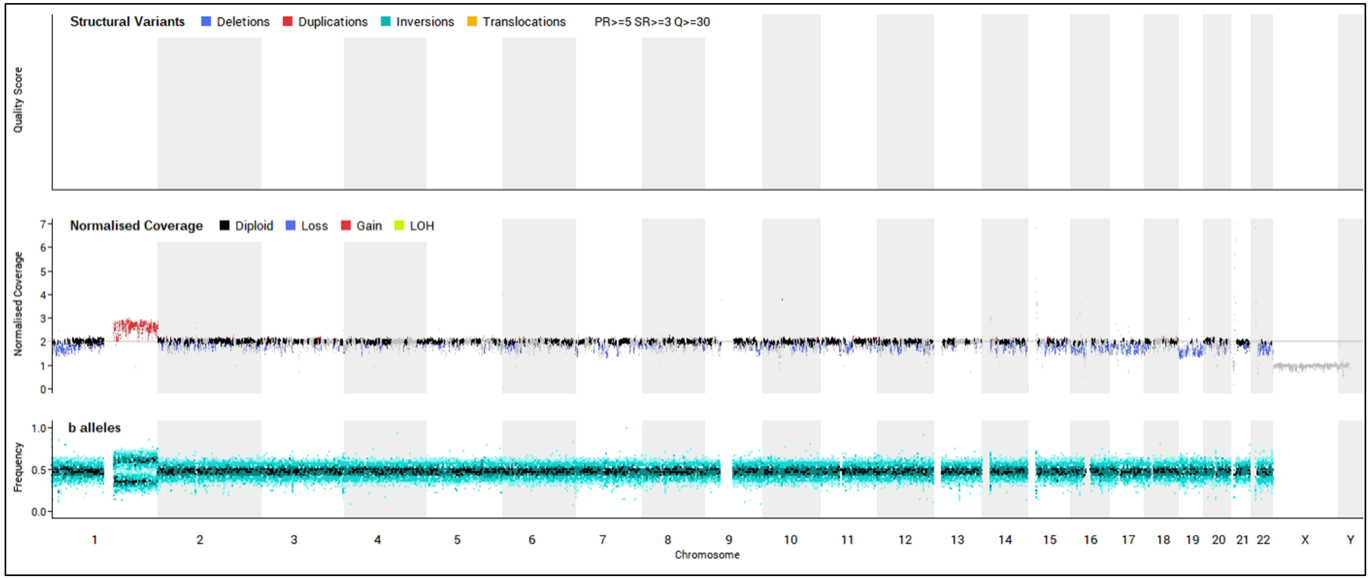

P2766 | Neuroblastoma (NB)

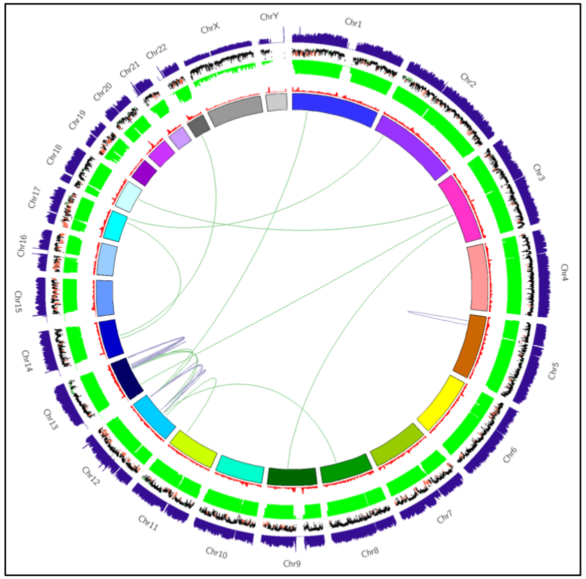

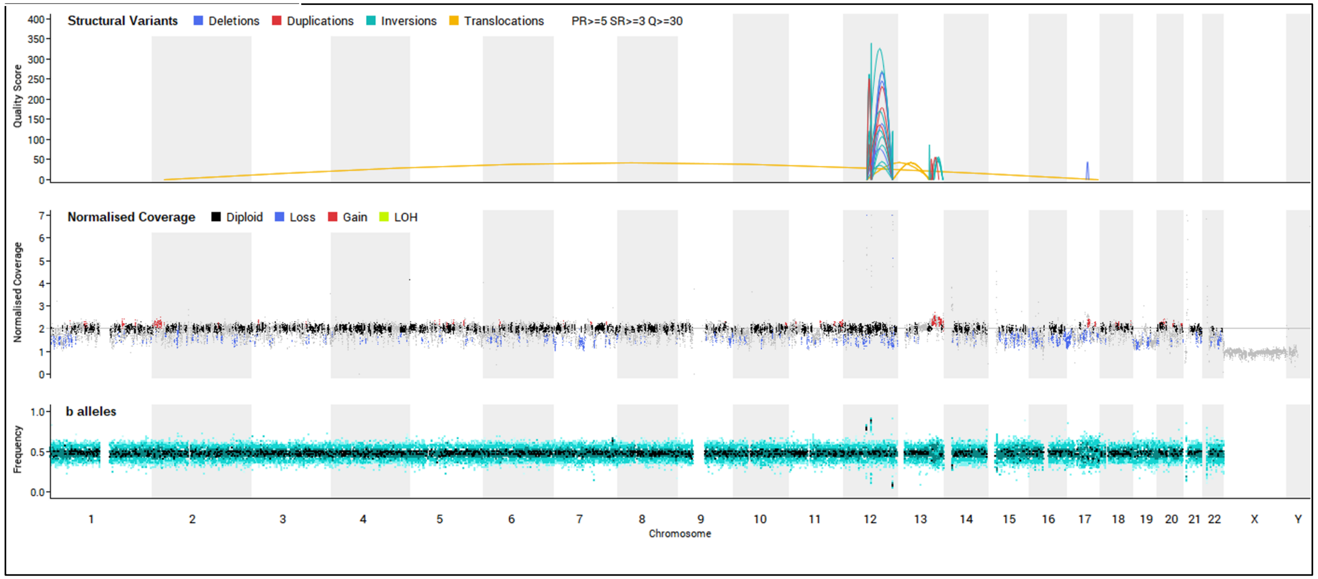

P2774 | Neuroblastoma (NB)

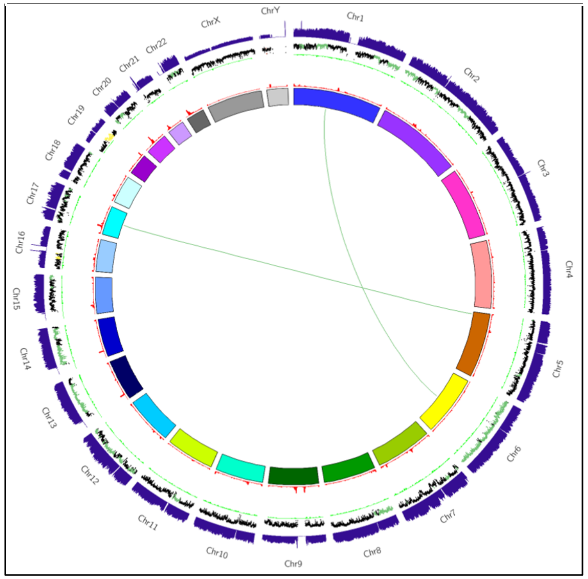

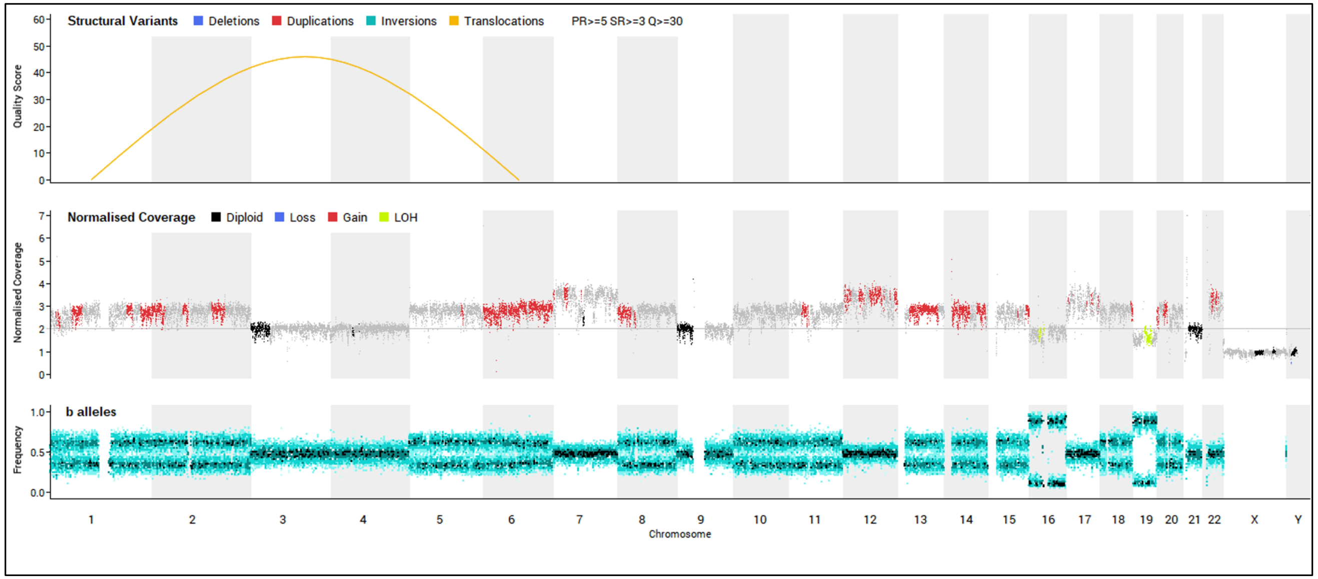

P3089 | Ganglio-neuroblastoma (G-NB)

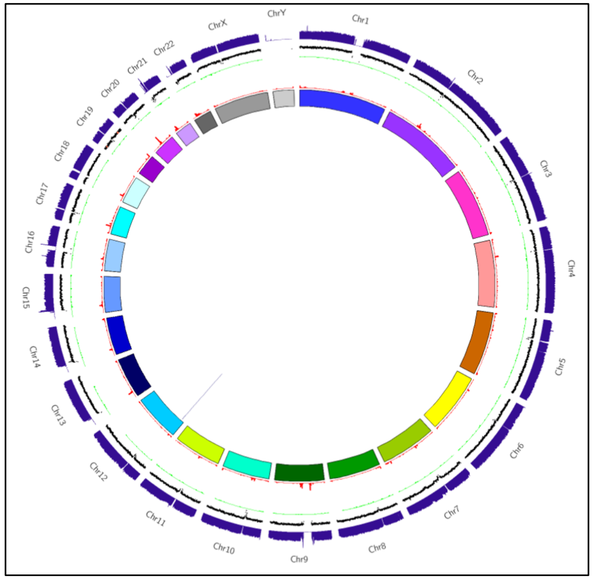

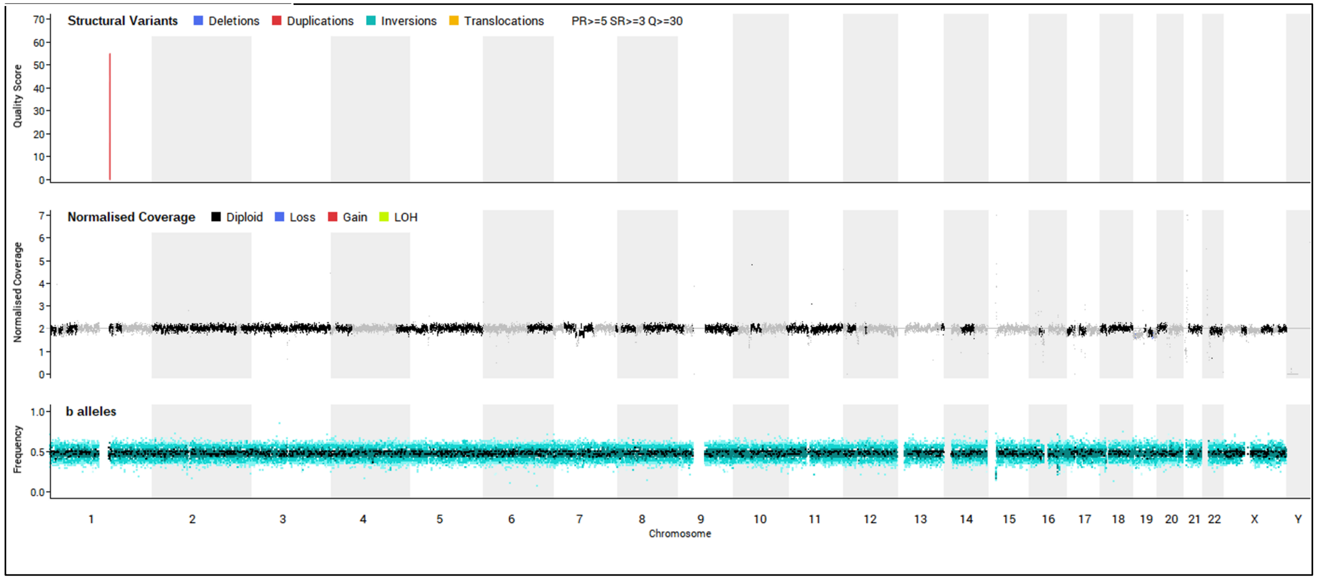

P3072 | Wilms' tumour (WT)

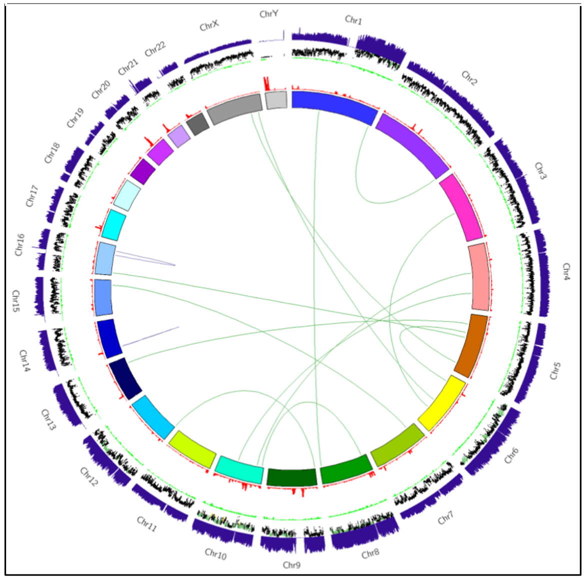

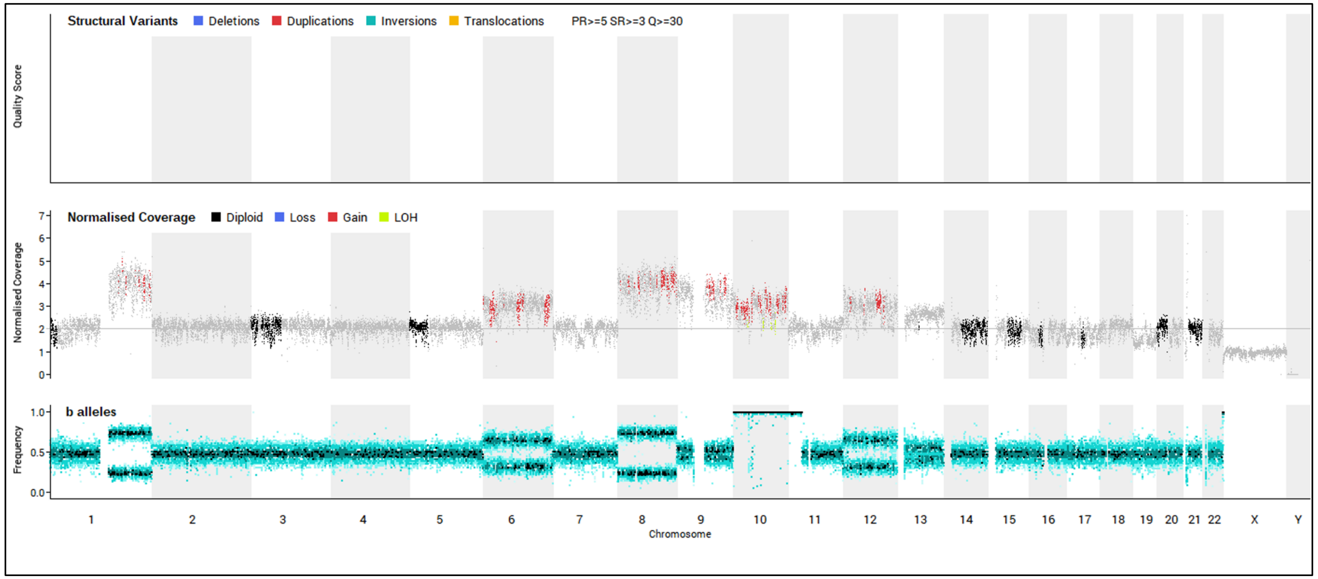

P3091 | Wilms' tumour (WT)

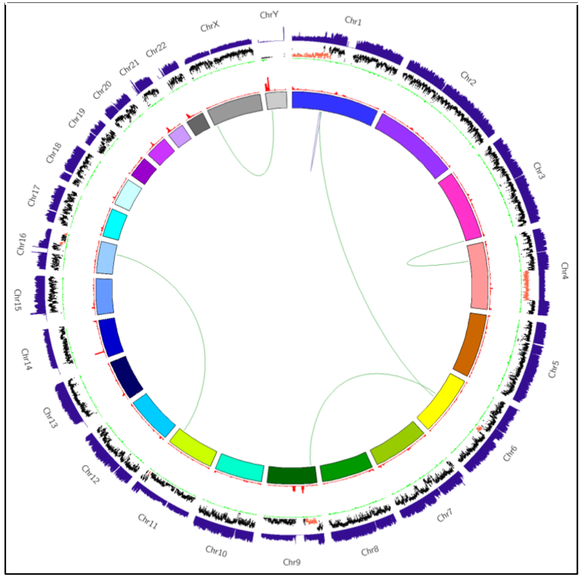

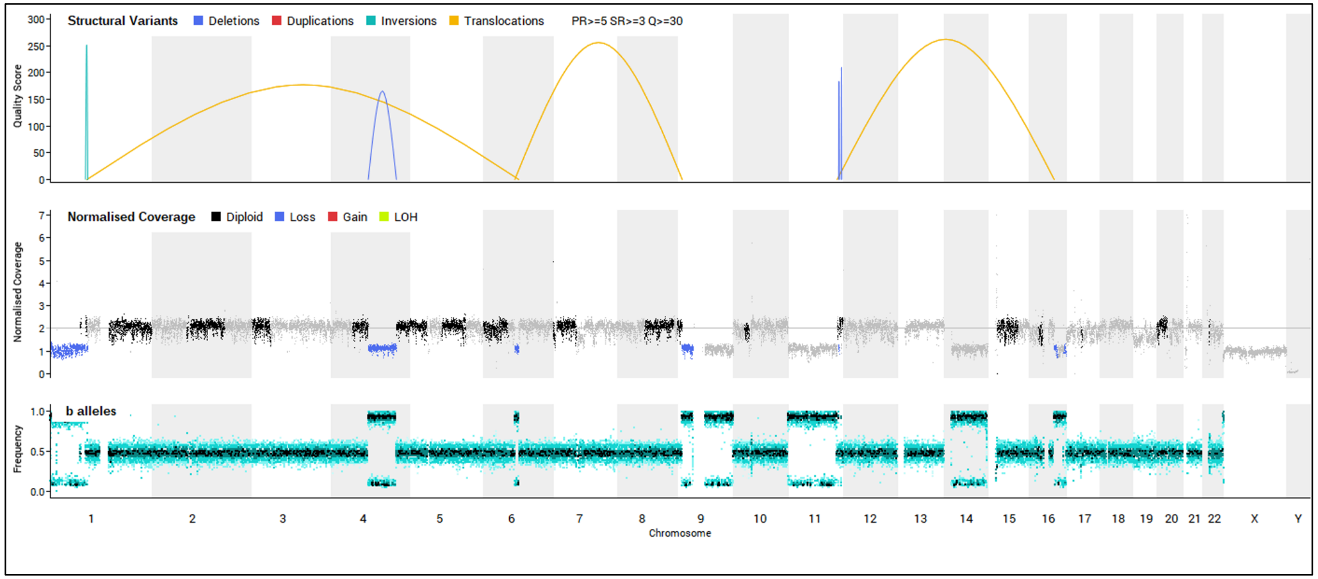

P2994 | Renal cell carcinoma (RCC)

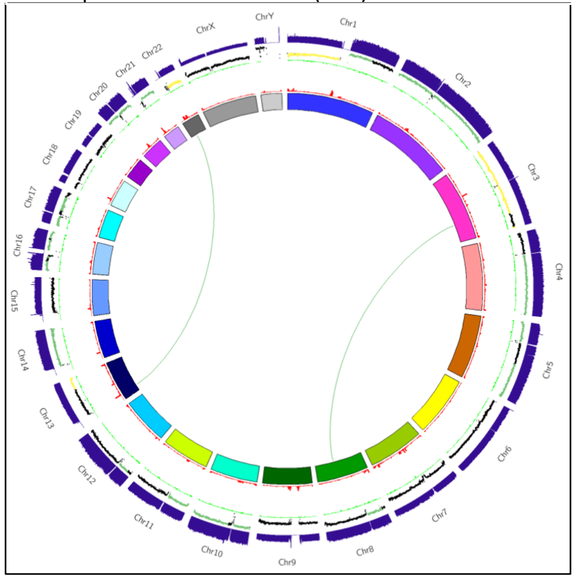

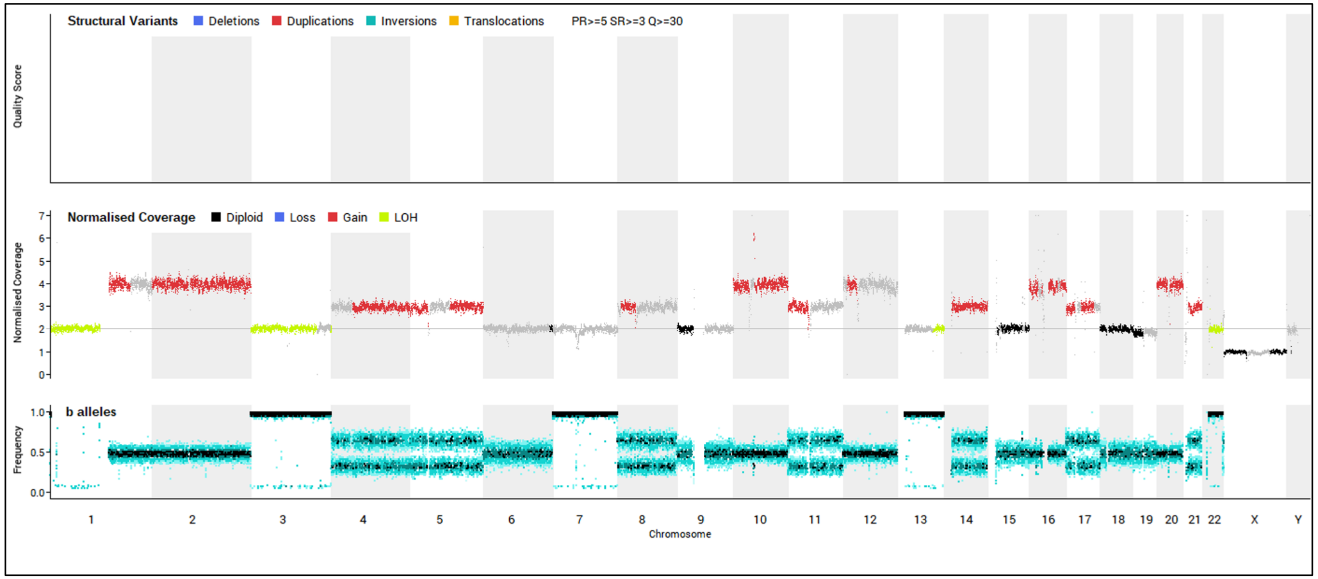

P2337 | Rhabdomyosarcoma (RMS)

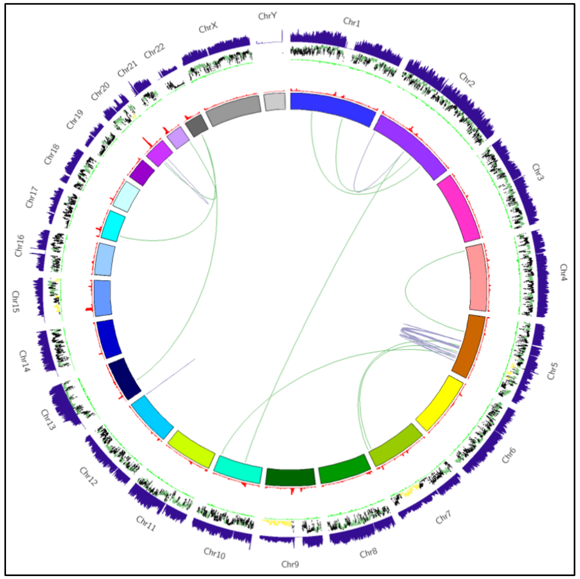

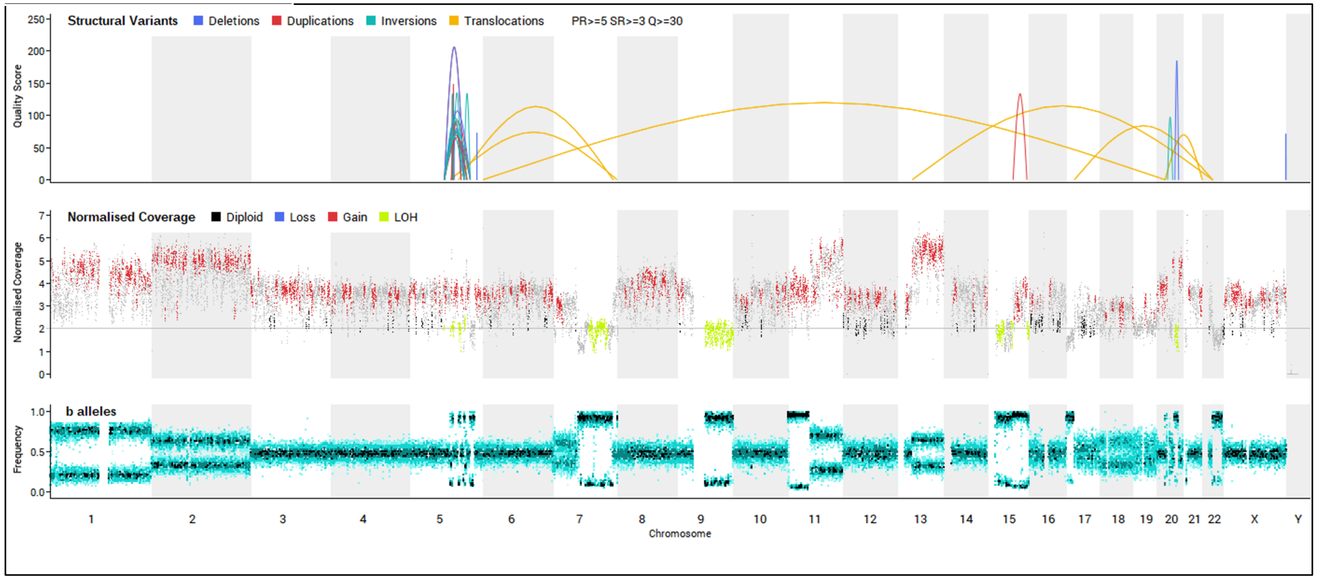

P2626 | Rhabdomyosarcoma (RMS)

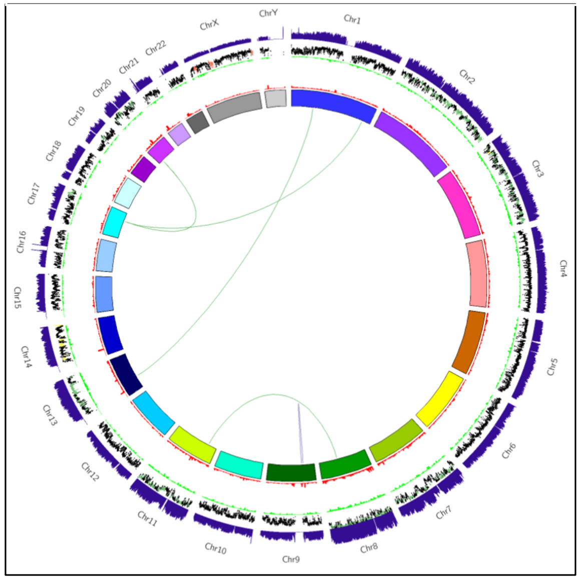

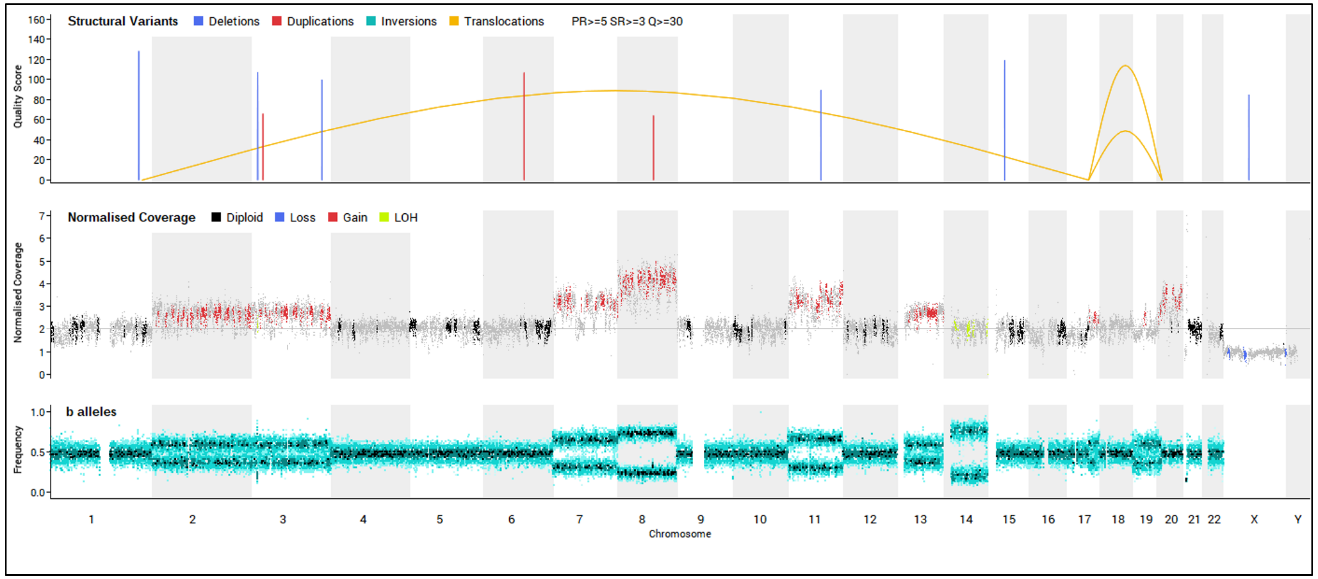

P2878 | Rhabdomyosarcoma (RMS)

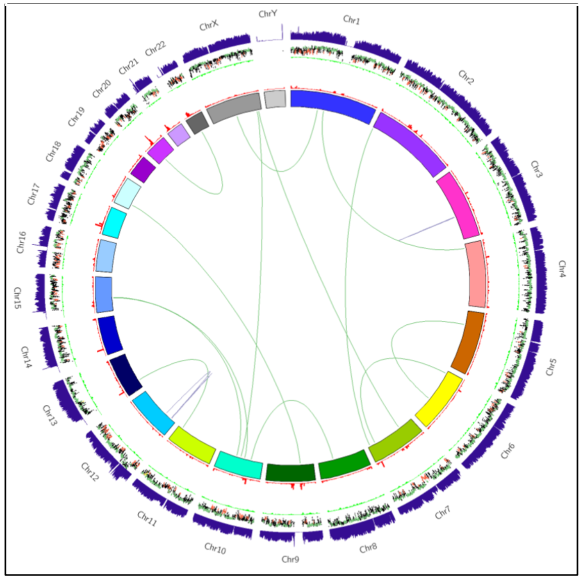

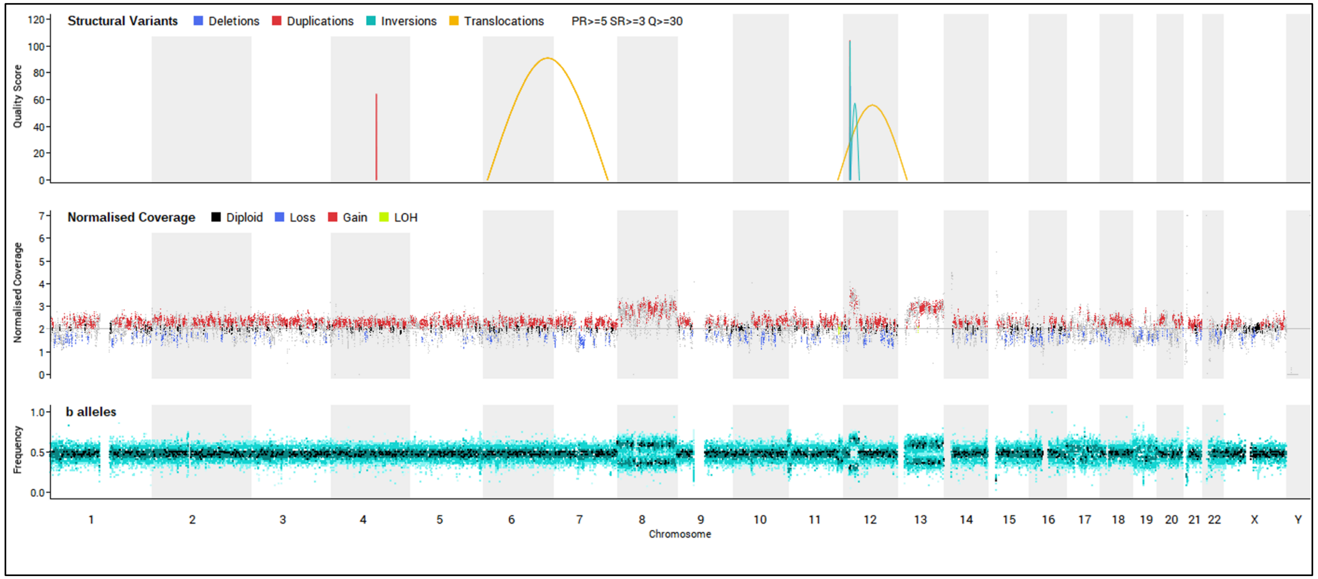

P3153 | Undifferentiated sarcoma (US)

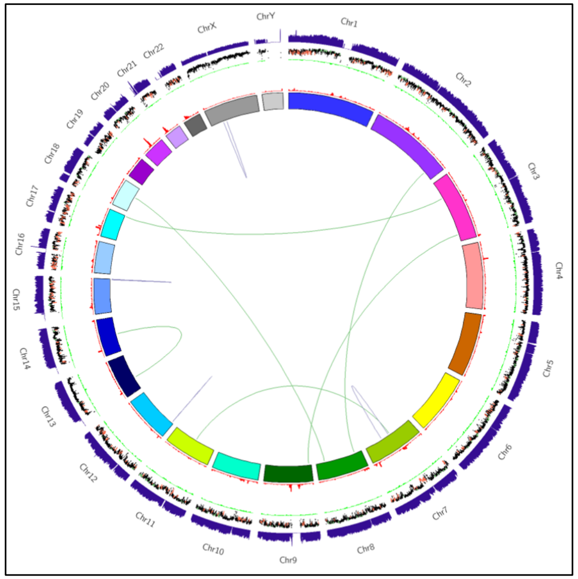

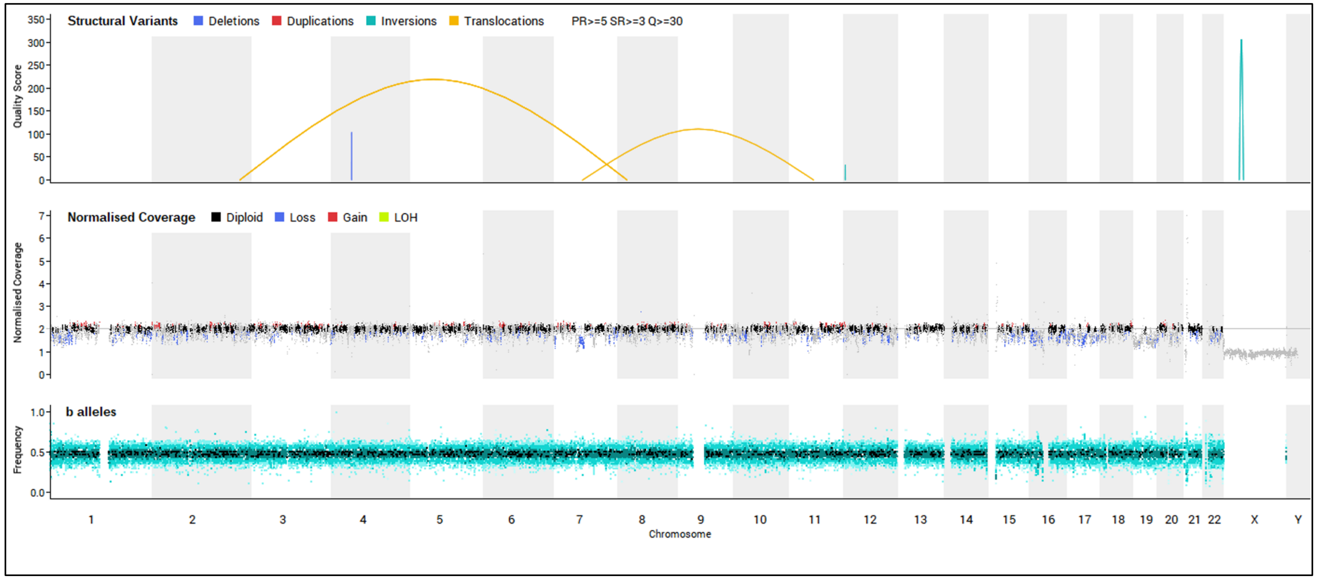

P2720 | Ewing's sarcoma (ES)

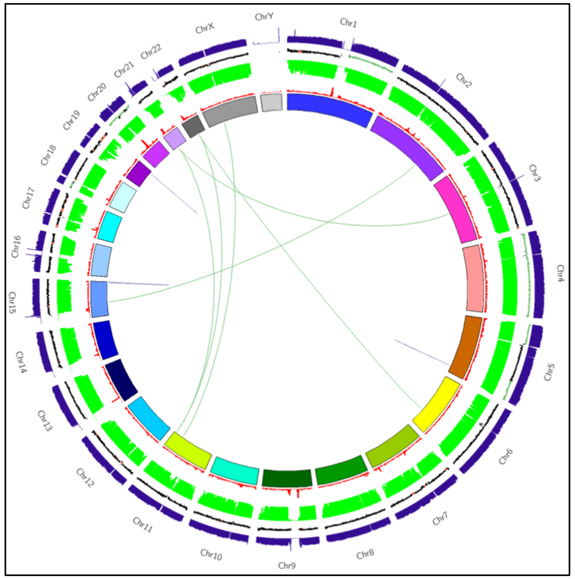

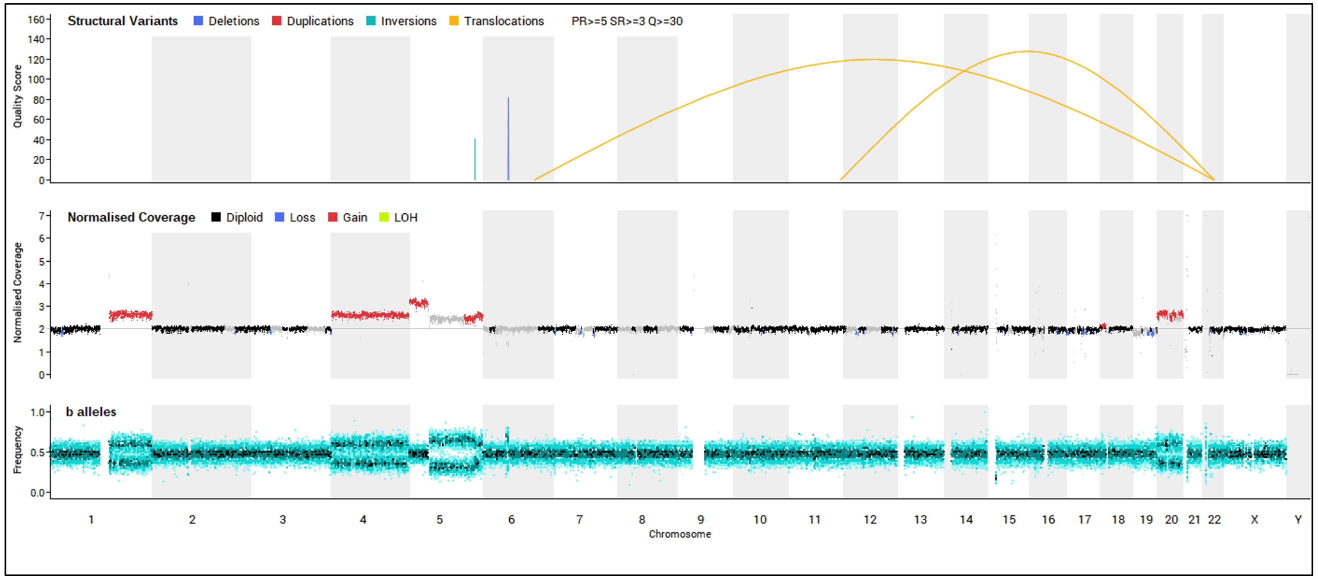

P3053 | Osteosarcoma (OS)

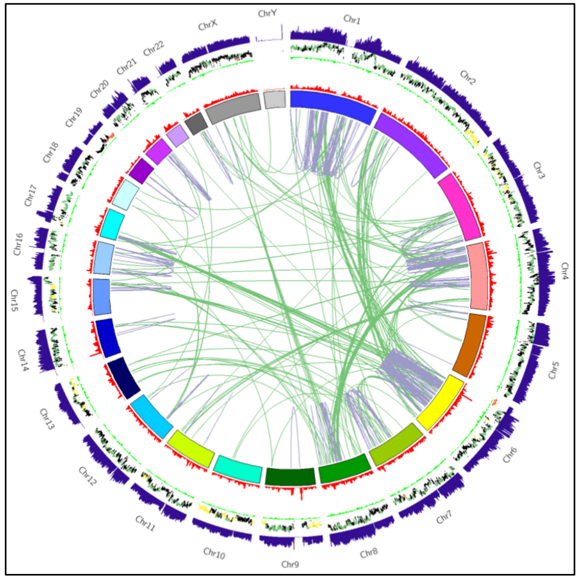

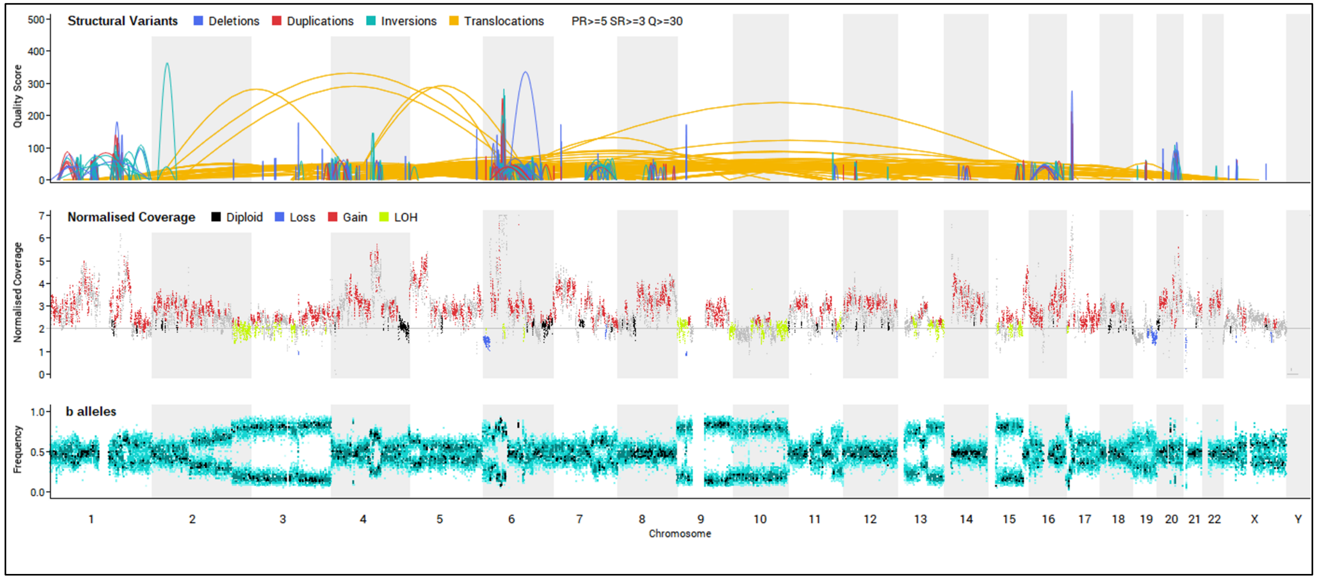

P2625 | Congenital infantile fibrosarcoma (CIFS)

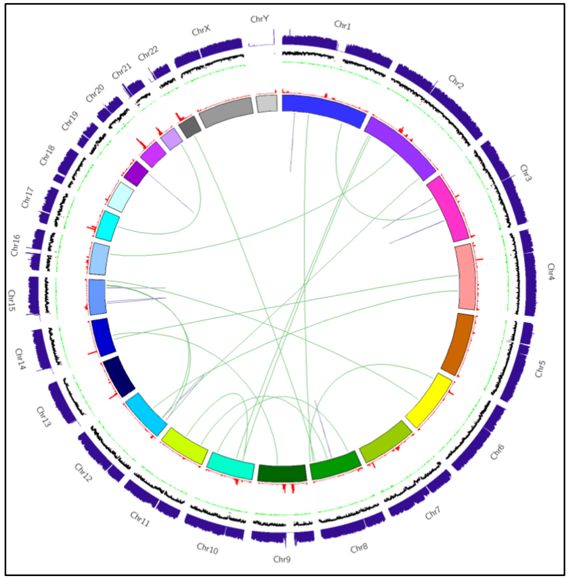

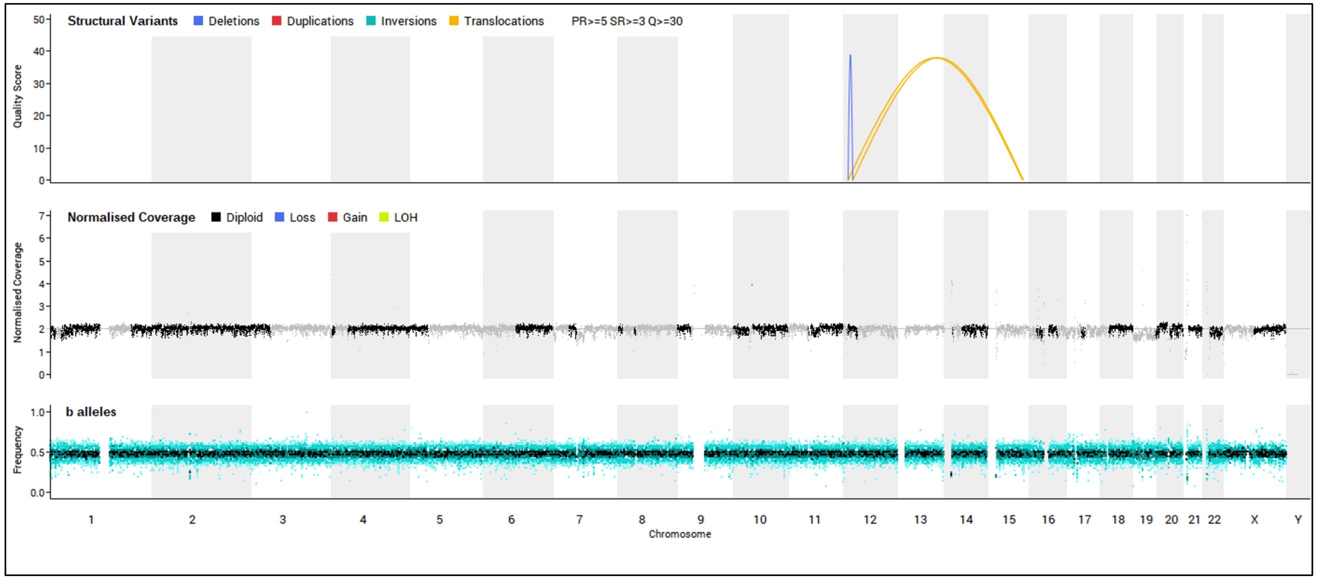

P3221 | Immature teratoma (IT)

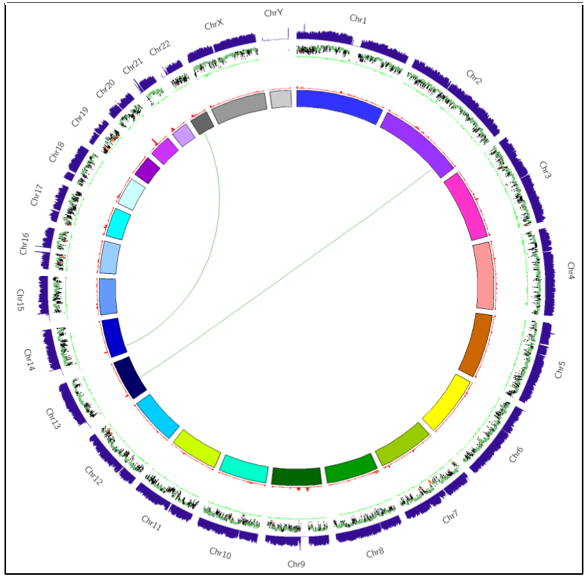

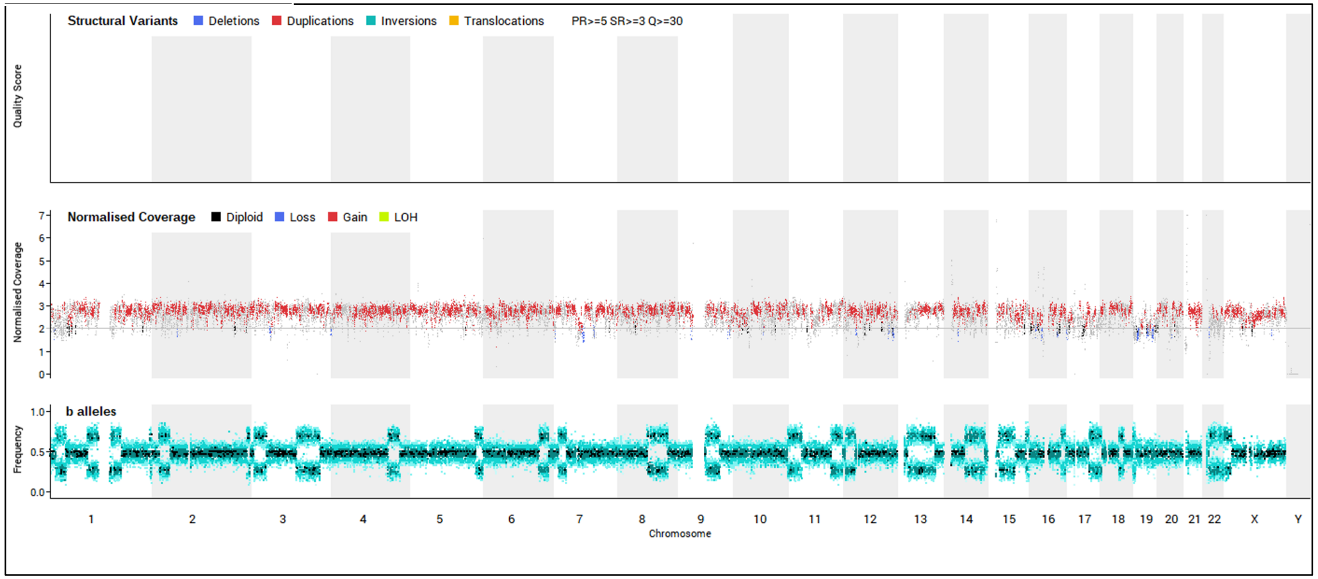

P2571 | Ovarian granulosa cell tumour (OV_GRAN)

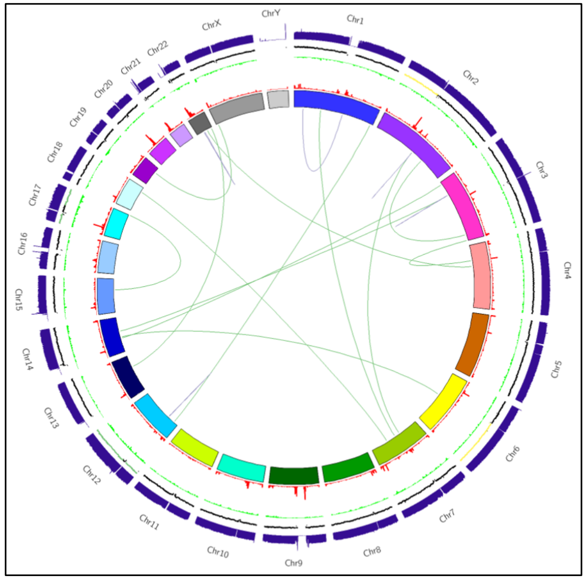

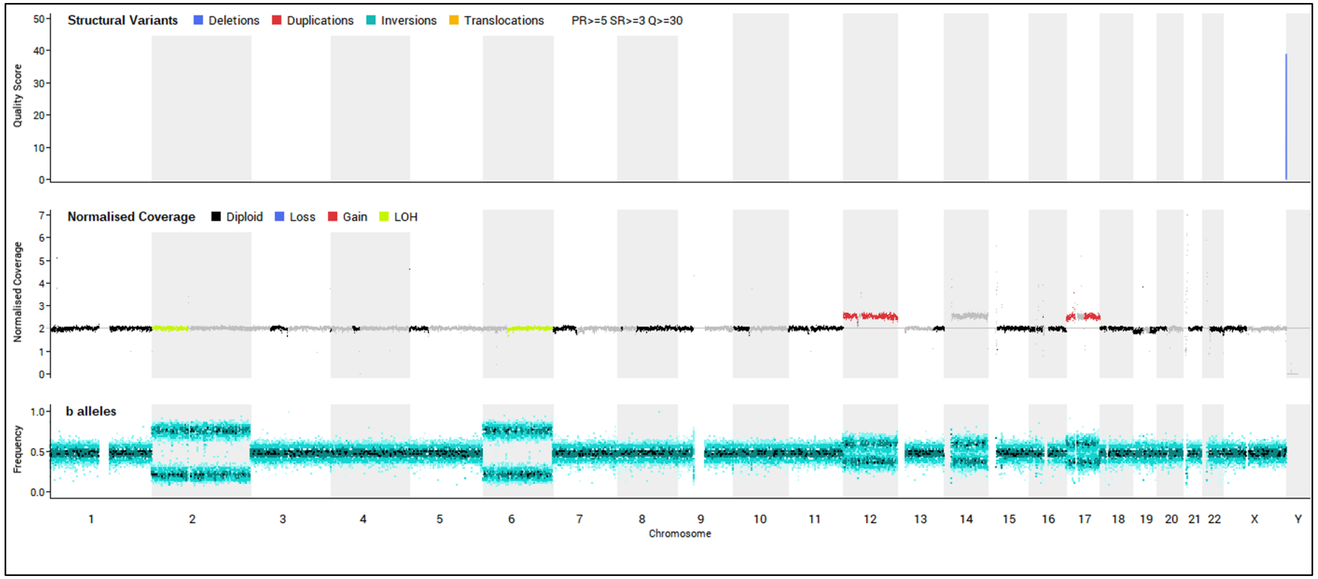

P3094 | High grade B-cell lymphoma (LYM)

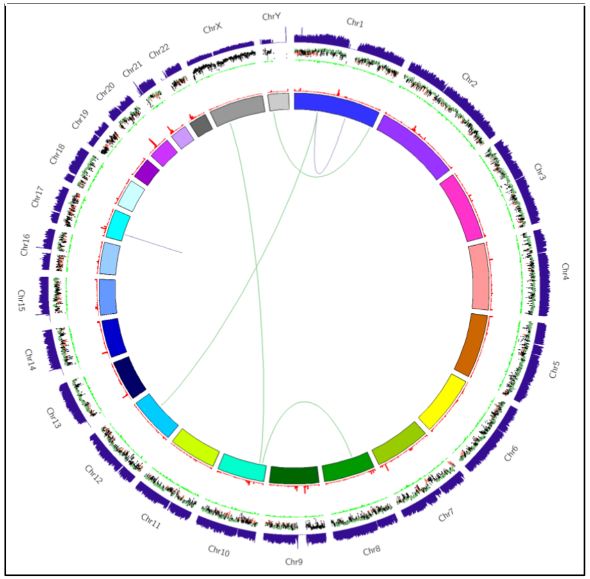

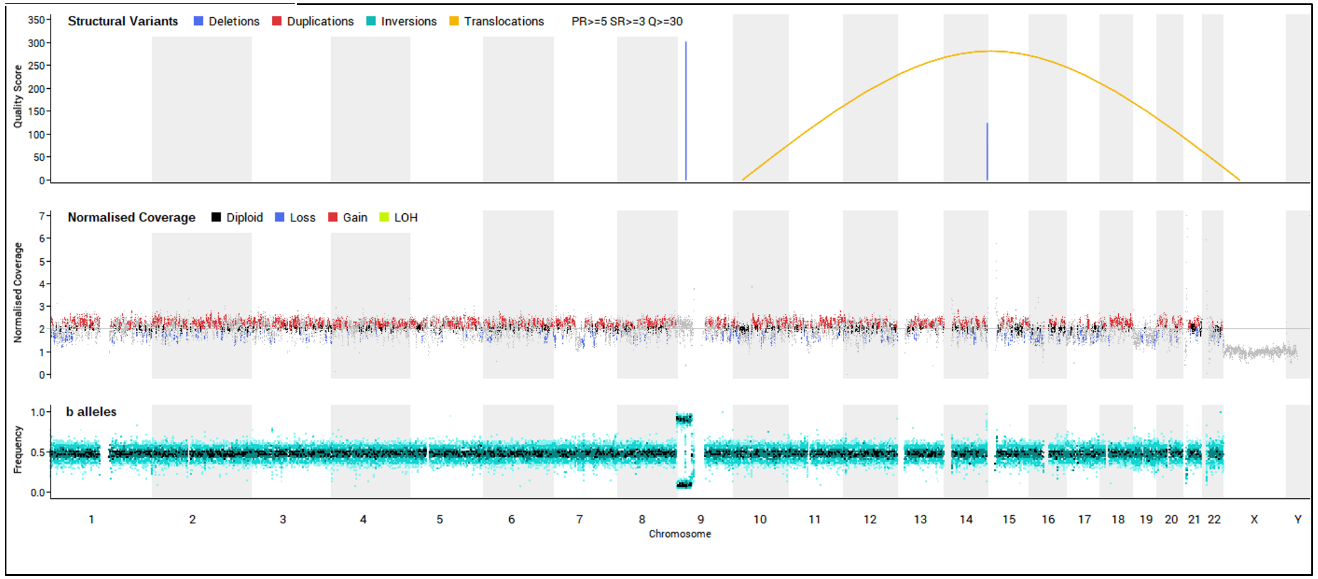

Supplement: Supplementary file 2 — Supplementary Figure 1 [file 41416_2022_1788_MOESM2_ESM.pdf]
