# Supplementary material for: The NHS England 100,000 Genomes Project: feasibility and utility of centralised genome sequencing for children with cancer
Source: Br J Cancer. 2022 Apr 22;127(1):137–44. doi: 10.1038/s41416-022-01788-5 (PMC9276782; doi:10.1038/s41416-022-01788-5)
Supplement: Supplementary file 3 — Supplementary Table 1 [file 41416_2022_1788_MOESM3_ESM.pdf]

Patient Details							Assay Details							Pre-Standard of Care Investigations		Additional to SOC	Cinical Value				Germline PanelApp Panels Applied							
Sample Identifier	Sex	Age at Diagnosis (Yr)	Tissue	Diagnosis	Revised / Refined Diagnosis	Cancer Status	Tumour content	Library	Tumour coverage	Normal Coverage	Somatic SNVs	Somatic Indels	Somatic SVs	Genes interogate by SOC (-ve result) Somatic and [Germline]	Genes interogate by SOC (+ve result) Somatic and [Germline]	Variants unique to WGS	Diagnosis	Informs prognosis	Therapeutic opportunity (Potential Drug)	Germline	Genomics England https://www.genomicsengland.co.uk/about-genomics-england/the-	Childhood solid tumours https://panelapp.genomicsengland.co.uk/panels/259/	Adult solid tumours https://panelapp.genomicsengland.co.uk/panels/245/	Ovarian cancer https://panelapp.genomicsengland.co.uk/panels/117/	Renal cancer https://panelapp.genomicsengland.co.uk/panels/154/	Haematological malignancies https://panelapp.genomicsengland.co.uk/panels/59/	Sarcoma https://panelapp.genomicsengland.co.uk/panels/217/	Thyroid cancer https://panelapp.genomicsengland.co.uk/panels/421/
P2624	Female	2	CNS	Medulloblastoma (MB) - SHH-activated	MYCN-activated	Primary	High >60%	FF PCR-free	106.69	32.61	4179	984	35	MYC, MYCN, TP53		SUFU, SUFU, MYCN, MAX	Refine (MYCN)	MYCN	-			1	1					
P2803	Male	5.2	CNS	Medulloblastoma (MB) - classical, WNT-negative	SHH-activated	Primary	High >60%	FF PCR-free	103.1	51.91	7833	1207	32	MYC		SUFU, MAP2K4	Refine (SUFU)	SUFU	-			1	1					
P2981	Male	5.2	CNS	Medulloblastoma (MB) - non-WNT/non-SHH (group 4)	-	Primary	High >60%	FF PCR-free	88.96	41.9	3609	884	19	MYC, MYCN, SMARCA4, H3F3A			Uninformative	-	-			1	1					
P2887	Male	5.2	CNS	Medulloblastoma (MB) - non-WNT/non-SHH (group 4)	-	Primary	High >60%	FF PCR-free	104.08	30.41	4751	936	41	MYC, MYCN, SMARCA4			Uninformative	-	-			1	1					
P2801	Male	3.6	CNS	Medulloblastoma (MB) - myoblastic differentiation (group 3)	-	Primary	High >60%	FF PCR-free	113.97	31.38	5664	1761	49	MYC, MYCN, SMARCA4			Uninformative	-	-			1	1					
P2955	Female	2	CNS	Anaplastic ependymoma (EP)	-	Primary	High >60%	FF PCR-free	104.74	31	6701	9666	34	[NF1, SPRED1, GNAS], MLH1, MSH2, MSH6	[PMS2]	[PMS2]	Consistent	[PMS2]	Hypermutation (e.g. Pembrolizumab)	[PMS2] [PMS2]		1	1					
P2767	Male	5.5	CNS	Pineoblastoma (PB)	-	Primary	High >60%	FF Nano (PCR)	103.68	48.14	11397	71108	122	H3F3A		PIK3CA, CTNNB1	Uninformative	-	PIK3CA (e.g. Alpelisib, Fulvestrant)			1	1					
P2847	Male	10.8	CNS	Biphasic neuroepithelial tumour (LGG/HGG)	-	Primary	Medium 40-60%	FF PCR-free	104.82	29.56	5572	680	101	BRAF, CDK4, EGFR, PDGFRA, MYCN, H3F3A		CDKN2A, ZNF394-BRAF	Consistent	-	BRAF (e.g. Trametinib)			1	1					
P2806	Male	1.4	CNS	Pilocytic astrocytoma (PA)	-	Recurrence	High >60%	FF PCR-free	93.76	29.83	3742	640	21	[MLH1, MSH2, MSH6, PMS2], H3F3A, BRAF	[NF1]	NF1	Consistent	-	NF1 (e.g. Cobimetinib, Trametinib)	[NF1]		1	1					
P2831	Male	3.8	CNS	Pilocytic astrocytoma (PA)	-	Primary	High >60%	FF PCR-free	100.85	52.27	2216	406	5	H3F3A, BRAF	KIAA1549-BRAF		Consistent	-	BRAF (e.g. Trametinib)			1	1					
P2830	Female	1	CNS	Glioma with molecular features of pleomorphic xanthoastrocytoma (PXA)	Infant-type hemispheric glioma	Primary	High >60%	FF PCR-free	94.18	37.44	3424	757	20	KIAA1549-BRAF, IDH1		CCDC88A-ALK	Modify (CCDC88A-ALK)	ALK	ALK (e.g. Crizotinib)			1	1					
P3088	Male	9.5	CNS	Diffuse Leptomeningeal Glioneuronal Tumour (DLGNT)	-	Metastasis	Medium 40-60%	FF PCR-free	102.46	40.14	4392	1376	15	IDH1, BRAF		FGFR1, PTPN11	Consistent	-	FGFR1 (e.g. Erdafitinib)			1	1					
P2627	Male	7.6	CNS	Dysembryoplastic neuroepithelial tumour (DNET)	-	Primary	Medium 40-60%	FF PCR-free	99.91	50.59	3040	509	10	IDH1, IDH2, BRAF, ATRX, MLH1, MSH2, MSH6, PMS2		FGFR1	Consistent	-	FGFR1 (e.g. Erdafitinib)			1	1					
P2058	Female	14	CNS	Astroblastoma (AB)	-	Primary	High >60%	FF PCR-free	110.12	35.41	6218	1214	721	CIC, BCOR		MN1-GTSE1, EWSR1-PATZ1	Consistent	MN1	-		1							
P3269	Male	0.7	Adrenal	Adrenocortical carcinoma (ACC)	-	Primary	High >60%	FF PCR-free	103.08	53.62	3343	536	9	Multi-Gene NGS Panel	[11p UPD], CTNNB1, GNAS		Consistent	-	-	[11p UPD]		1	1					1
P3311	Female	3	Adrenal	Adrenocortical carcinoma (ACC)	-	Primary	High >60%	FF PCR-free	86.89	56.5	6270	789	29	MLH1, MSH2, MSH6, PMS2	CTNNB1	SDHA	Consistent	-	-			1	1					
P2623	Male	7.4	Liver	Hepatoblastoma (HB)	-	Primary	High >60%	FF Nano (PCR)	114.25	53.58	13915	98182	116	Multi-Gene NGS Panel	CTNNB1, TERT		Consistent	-	-			1	1					
P3244	Male	0.2	Liver	Hepatoblastoma (HB)	-	Recurrence	High >60%	FF Nano (PCR)	121.36	53.41	13080	103483	108	Chromsome Breakage Studies		CTNNB1	Consistent	-	-			1	1					
P3038	Female	1	Liver	Hepatoblastoma (HB)	-	Primary	High >60%	FF Nano (PCR)	112.08	52.77	12019	105308	94	[APC]		APC	Consistent	-	-			1	1					
P3155	Male	0.2	Liver	Hepatoblastoma (HB)	-	Primary	High >60%	FF PCR-free	97.1	35.67	3972	614	17				Uninformative	-	-			1	1					
P2766	Male	3.9	PNS	Neuroblastoma (NB)	-	Metastasis	High >60%	FF Nano (PCR)	117.6	49.79	14392	87972	196	ATM, MYCN, ALK		MDM2	Consistent	MDM2	-			1	1					
P2774	Male	0.9	PNS	Neuroblastoma (NB)	-	Primary	High >60%	FF PCR-free	101.23	53.33	3457	625	5	ATM, MYCN, ALK		SMARCA4	Consistent	SMARCA4	-			1	1					
P3089	Female	4	PNS	Ganglio-neuroblastoma (G-NB)	-	Primary	High >60%	FF PCR-free	100.57	49.58	3030	325	13	ATM, MYCN			Uninformative	-	-			1	1					
P3072	Male	15.1	Renal	Wilms' tumour (WT)	-	Metastasis	High >60%	FF PCR-free	99.63	30.75	5663	1136	33	Multi-Gene NGS Panel		MYCN, NONO, ASXL1	Consistent	-	-				1		1			
P3091	Male	3.3	Renal	Wilms' tumour (WT)	-	Primary	High >60%	FF PCR-free	96.61	53.84	3612	693	15			CTNNB1	Consistent	-	-			1	1					
P2994	Male	11.0	Renal	Renal cell carcinoma (RCC)	Wilms like tumour	Primary	High >60%	FF PCR-free	108.13	63.1	4505	675	21	[11p UPD], SS18, Multi-Gene NGS Panel	CTNNB1, KRAS	11p LOH	Modify (CTNNB1, KRAS, 11p LOH)	-	-			1	1					
P2337	Female	1	Sarcoma	Rhabdomyosarcoma (RMS)	-	Primary	High >60%	FF PCR-free	94.02	30.74	4789	852	67	[TP53], FOXO1, PAX3, PAX7		NRAS, TP53	Consistent	-	-			1	1					
P2626	Male	9.8	Sarcoma	Rhabdomyosarcoma (RMS)	-	Primary	High >60%	FF PCR-free	97.01	49.79	6901	1881	16	FOXO1		CTNNB1	Consistent	-	-			1	1					
P2878	Female	2	Sarcoma	Rhabdomyosarcoma (RMS)	-	Primary	High >60%	FF PCR-free	96.16	28.21	4381	856	32	FOXO1, PAX3, PAX7, ALK		KRAS	Consistent	-	-			1	1					
P3153	Male	16.2	Sarcoma	Undifferentiated sarcoma (US)	BCOR-CCNB3 sarcoma	Primary	High >60%	FF PCR-free	87.51	30.33	3961	727	20			BCOR-CCNB3	Modify (BCOR-CCNB3)	BCOR	-				1				1	
P2720	Female	12	Sarcoma	Ewing's sarcoma (ES)	-	Primary	High >60%	FF Nano (PCR)	85.59	44.02	9979	60810	81	Multi-Gene NGS Panel	EWSR1-FLI1		Consistent	-	-			1	1					
P3053	Female	14	Sarcoma	Osteosarcoma (OS)	-	Primary	High >60%	FF PCR-free	98.31	31.18	20836	1548	695	Multi-Gene NGS Panel	PDGFRA	TP53, CDKN2A	Consistent	-	Hypermutation (e.g. Pembrolizumab)			1	1					
P2625	Female	0	Sarcoma	Congenital infantile fibrosarcoma (CIFS)	-	Primary	High >60%	FF PCR-free	114.24	28.62	5029	708	60	Multi-Gene NGS Panel	ETV6-NTRK3		Consistent	-	NTRK (e.g. Larotrectinib)			1	1					
P3221	Female	13	Teratoma	Immature teratoma (IT)	-	Primary	High >60%	FF PCR-free	92.54	53.61	2363	449	7				Uninformative	-	-			1	1					
P2571	Female	2	Ovarian	Ovarian granulosa cell tumour (OV_GRAN)	-	Primary	High >60%	FF PCR-free	95.37	28.85	7901	1424	58	Multi-Gene NGS Panel			Uninformative	-	-		1			1				
P3094	Male	5.7	Lymphatic	High grade B-cell lymphoma (LYM)	MLLT10-DDX3X mutated lymphoma	Primary	High >60%	FF PCR-free	94.49	30.85	4889	1523	20	MYC, MLL, BCL6, BCL2, IGH, BCR-ABL1, ETV6-RUNX1	CDKN2A	IGH, KMT2D, MLLT10-DDX3X	Modify (MLLT10-DDX3X)	-	-			1	1			1		
