# Supplementary material for: The NHS England 100,000 Genomes Project: feasibility and utility of centralised genome sequencing for children with cancer
Source: Br J Cancer. 2022 Apr 22;127(1):137–44. doi: 10.1038/s41416-022-01788-5 (PMC9276782; doi:10.1038/s41416-022-01788-5)
Supplement: Supplementary file 4 — Supplementary Table 2 [file 41416_2022_1788_MOESM4_ESM.pdf]

Sample Identifier	Tissue	Cancer Diagnosis	Gene	Transcript	Consequence	Domain	GRCh38 Position	HGVS Annotation	VCF Filter Status	Variant Clinical Status	Reportable Genes	Diagnosis	Informs prognosis	Therapeutic opportunity	Germline
P2571	Ovarian	Ovarian granulosa cell tumour (OV_GRAI	-	-	-	-	-	-	-	-	No reportable variants detected	Uninformative	-	-	-
P2801	CNS	Medulloblastoma (MB)	-	-	-	-	-	-	-	-	No reportable variants detected	Uninformative	-	-	-
P2887	CNS	Medulloblastoma (MB)	-	-	-	-	-	-	-	-	No reportable variants detected	Uninformative	-	-	-
P2981	CNS	Medulloblastoma (MB)	-	-	-	-	-	-	-	-	No reportable variants detected	Uninformative	-	-	-
P3089	PNS	Ganglio-neuroblastoma (G-NB)	-	-	-	-	-	-	-	-	No reportable variants detected	Uninformative	-	-	-
P3155	Liver	Hepatoblastoma (HB)	-	-	-	-	-	-	-	-	No reportable variants detected	Uninformative	-	-	-
P3221	Teratoma	Immature teratoma (IT)	-	-	-	-	-	-	-	-	No reportable variants detected	Uninformative	-	-	-
P2994	Renal	Renal cell carcinoma (RCC)	11p LOH		LOH	Domain_1	-	-	PASS	Novel	CTNNB1, KRAS,11p LOH	Modify (11pLOH)	-	-	-
P3038	Liver	Hepatoblastoma (HB)	APC	ENST00000508376	Deletion	-	5:92239122-135117383	-	NON_PASS	Novel	APC	Consistent	-	-	-
P3072	Renal	Wilms' tumour (WT)	ASXL1	ENST00000375687	Substitution	Domain_2	20:32435605C>T	c.2893C>T p.(Arg965*)	PASS	Novel	MYCN, NONO, ASXL1	Consistent	-	-	-
P3153	Sarcoma	Undifferentiated sarcoma (US)	BCOR-CCNB3	-	Fusion	-	X:40052040-50307090	-	PASS	Novel	BCOR-CCNB3	Modify (BCOR-CCNB3)	BCOR	-	-
P2830	CNS	Glioma with molecular features of pleomo	CCDC88A-ALK	-	Fusion	-	2:29223670-55342739	-	NON_PASS	Novel	CCDC88A-ALK	Modify (CCDC88A-ALK)	ALK	ALK	-
P2847	CNS	Biphasic neuroepithelial tumour (LGG/HG	CDKN2A	ENST00000304494	Deletion	-	9:21132150-222910369	-	PASS	Novel	CDKN2A, ZNF394-BRAF	Consistent	-	-	-
P3053	Sarcoma	Osteosarcoma (OS)	CDKN2A	ENST00000304494	Deletion	-	9:21242198-24175397	-	PASS	Novel	PDGFRA, TP53, CDKN2A	Consistent	-	Hypermutation (Check	-
P3094	Lymphatic	High grade B-cell lymphoma (LYM)	CDKN2A	ENST00000304494	Deletion	-	9:21560655-22273874	-	NON_PASS	Known via SOC	CDKN2A, IGH, KMT2D, MLLT10-DI	Uninformative	-	-	-
P2623	Liver	Hepatoblastoma (HB)	CTNNB1	ENST00000349496	Deletion	-	3:41224361-41224696	-	PASS	Known via SOC	CTNNB1, TERT	Consistent	-	-	-
P2626	Sarcoma	Rhabdomyosarcoma (RMS)	CTNNB1	ENST00000349496	Substitution	Domain_1	3:41233420T>A	c.1161T>A p.(Asn387Lys)	PASS	Novel	CTNNB1	Consistent	-	-	-
P3091	Renal	Wilms' tumour (WT)	CTNNB1	ENST00000349496	Deletion	-	3:41208648-41224862	-	NON_PASS	Novel	CTNNB1	Consistent	-	-	-
P3244	Liver	Hepatoblastoma (HB)	CTNNB1	ENST00000349496	Deletion	-	3:41224615-41225154	-	PASS	Novel	CTNNB1	Consistent	-	-	-
P3269	Adrenal	Adrenocortical carcinoma (ACC)	CTNNB1	ENST00000349496	Substitution	Domain_1	3:41224622C>G	c.110C>G p.(Ser37Cys)	PASS	Known via SOC	UPD11p, CTNNB1, GNAS	Consistent	-	-	-
P3311	Adrenal	Adrenocortical carcinoma (ACC)	CTNNB1	ENST00000349496	Substitution	Domain_1	3:41224609T>C	c.97T>C p.(Ser33Pro)	PASS	Known via SOC	CTNNB1, SDHA	Consistent	-	-	-
P2994	Renal	Renal cell carcinoma (RCC)	CTNNB1	ENST00000349496	Substitution	Domain_1	3:41224646C>T	c.134C>T p.(Ser45Phe)	PASS	Known via SOC	CTNNB1, KRAS,11p LOH	Modify	-	-	-
P2767	CNS	Pineoblastoma (PB)	CTNNB1	ENST00000349496	Substitution	Domain_1	3:41224622C>T	c.110C>T p.(Ser37Phe)	PASS	Novel	PIK3CA, CTNNB1	Uninformative	-	-	-
P2625	Sarcoma	Congenital infantile fibrosarcoma (CIFS)	ETV6-NTRK3	-	Fusion	-	12:11882710_15:84928258	-	PASS	Known via SOC	ETV6-NTRK3	Consistent	-	NTRK	-
P2720	Sarcoma	Ewing's sarcoma (ES)	EWSR1-FLI1	-	Fusion	-	11:128778132_22:2928970	-	PASS	Known via SOC	EWSR1-FLI1	Consistent	-	-	-
P2058	CNS	Astroblastoma (AB)	EWSR1-PATZ1	-	Fusion	-	22:29289499-31344625	-	PASS	Novel	MN1-GTSE1, EWSR1-PATZ1	Consistent	-	-	-
P2627	CNS	Dysembryoplastic neuroepithelial tumour	FGFR1	ENST00000447712	Tandem Duplication	-	8:38413635-38418644	-	PASS	Novel	FGFR1	Consistent	-	FGFR1	-
P3088	CNS	Diffuse Leptomeningeal Glioneuronal Tu	FGFR1	ENST00000447712	Substitution	Domain_1	8:38417333T>C	c.1636A>G p.(Asn546Asp)	PASS	Novel	FGFR1, PTPN11	Consistent	-	FGFR1	-
P3269	Adrenal	Adrenocortical carcinoma (ACC)	GNAS	ENST00000354359	Substitution	Domain_2	20:58909365C>T	c.604C>T p.(Arg202Cys)	PASS	Known via SOC	UPD11p, CTNNB1, GNAS	Consistent	-	-	-
P3094	Lymphatic	High grade B-cell lymphoma (LYM)	IGH		Deletion	-	14:105864257-106421708	-	NON_PASS	Novel	CDKN2A, IGH, KMT2D, MLLT10-DI	Uninformative	-	-	-
P2831	CNS	Pilocytic astrocytoma (PA)	KIAA1549-BRAF	-	Fusion	-	7:138865965-140787622	-	NON_PASS	Known via SOC	KIAA1549-BRAF	Consistent	-	BRAF	-
P3094	Lymphatic	High grade B-cell lymphoma (LYM)	KMT2D	ENST00000301067	Substitution	Domain_2	12:49034811C>T	c.10355+1G>A	PASS	Novel	CDKN2A, IGH, KMT2D, MLLT10-DI	Uninformative	-	-	-
P2878	Sarcoma	Rhabdomyosarcoma (RMS)	KRAS	ENST00000256078	Substitution	Domain_1	12:25245347C>T	c.38G>A p.(Gly13Asp)	PASS	Novel	KRAS	Consistent	-	-	-
P2994	Renal	Renal cell carcinoma (RCC)	KRAS	ENST00000256078	Substitution	Domain_1	12:25245350C>T	c.35G>A p.(Gly12Asp)	PASS	Known via SOC	CTNNB1, KRAS,11p LOH	Modify	-	-	-
P2803	CNS	Medulloblastoma (MB)	MAP2K4	ENST00000415385	Substitution	Domain_1	17:12095573A>G	c.427-2A>G	PASS	Novel	SUFU, MAP2K4	Consistent	-	-	-
P2624	CNS	Medulloblastoma (MB)	MAX	ENST00000358664	Substitution	Domain_2	14:65078029C>T	c.179G>A p.(Arg60Gln)	PASS	Novel	SUFU, SUFU, MYCN, MAX	Consistent	-	-	-
P2766	PNS	Neuroblastoma (NB)	MDM2	ENST00000258149	Amplification	-	12:6881392-68883550	-	NON_PASS	Novel	MDM2	Consistent	MDM2	-	-
P3094	Lymphatic	High grade B-cell lymphoma (LYM)	MLLT10-DDX3X	-	Fusion	-	10:21730565_X:41341628	-	PASS	Novel	CDKN2A, IGH, KMT2D, MLLT10-DI	Modify (MLLT10-DDX3X)	-	-	-
P2058	CNS	Astroblastoma (AB)	MN1-GTSE1	-	Fusion	-	22:27757259-46301569	-	PASS	Novel	MN1-GTSE1, EWSR1-PATZ1	Consistent	MN1	-	-
P3072	Renal	Wilms' tumour (WT)	MYCN	ENST00000281043	Substitution	Domain_2	2:15942195C>T	c.131C>T p.(Pro44Leu)	PASS	Novel	MYCN, NONO, ASXL1	Consistent	-	-	-
P2624	CNS	Medulloblastoma (MB)	MYCN	ENST00000281043	Substitution	Domain_2	2:15942195C>T	c.131C>T p.(Pro44Leu)	PASS	Novel	SUFU, SUFU, MYCN, MAX	Refine (MYCN)	MYCN	-	-
P2806	CNS	Pilocytic astrocytoma (PA)	NF1	ENST00000358273	Indel	Domain_1	17:31258419GC>G	c.4251delC	PASS	Novel	NF1, NF1	Consistent	-	NF1	-
P2806	CNS	Pilocytic astrocytoma (PA)	NF1	ENST00000358273	Indel	Tier 1 (Germline)	17:31230323AGTTG>TTCA	c.3054_3058del5ins26	PASS	Known via SOC	NF1, NF1	Consistent	-	NF1	NF1
P3072	Renal	Wilms' tumour (WT)	NONO	ENST00000276079	Substitution	Domain_2	X:71291848G>A	c.224G>A p.(Arg75His)	PASS	Novel	MYCN, NONO, ASXL1	Consistent	-	-	-
P2337	Sarcoma	Rhabdomyosarcoma (RMS)	NRAS	ENST00000369535	Substitution	Domain_1	1:114713909G>T	c.181C>A p.(Gln61Lys)	PASS	Novel	NRAS, TP53	Consistent	-	-	-
P3053	Sarcoma	Osteosarcoma (OS)	PDGFRA	ENST00000257290	Amplification	-	4:54238263-54290156	-	NON_PASS	Known via SOC	PDGFRA, TP53, CDKN2A	Consistent	-	Hypermutation (Check	-
P2767	CNS	Pineoblastoma (PB)	PIK3CA	ENST00000263967	Substitution	Domain_1	3:179203760G>A	c.1030G>A p.(Val344Met)	PASS	Novel	PIK3CA, CTNNB1	Uninformative	-	PIK3CA	-
P2955	CNS	Anaplastic ependymoma (EP)	PMS2	ENST00000265849	Indel	Tier 3 (Germline)	7:5986933A>AT	c.1831dupA	PASS	Novel	PMS2	Consistent	PMS2	Hypermutation (Check	PMS2
P3088	CNS	Diffuse Leptomeningeal Glioneuronal Tu	PTPN11	ENST00000351677	Substitution	Domain_1	12:112450385G>A	c.205G>A p.(Glu69Lys)	PASS	Novel	FGFR1, PTPN11	Consistent	-	-	-
P3311	Adrenal	Adrenocortical carcinoma (ACC)	SDHA	ENST00000264932	Substitution	Domain_2	5:240357G>A	c.1433-1G>A	PASS	Novel	CTNNB1, SDHA	Consistent	-	-	-
P2774	PNS	Neuroblastoma (NB)	SMARCA4	ENST00000450717	Substitution	Domain_1	19:10984335G>T	c.184G>T p.(Gly62*)	PASS	Novel	SMARCA4	Consistent	SMARCA4	-	-
P2624	CNS	Medulloblastoma (MB)	SUFU	ENST00000369902	Deletion	-	10:101786051-102894381	-	NON_PASS	Novel	SUFU, SUFU, MYCN, MAX	Consistent	-	-	-
P2624	CNS	Medulloblastoma (MB)	SUFU	ENST00000369902	Deletion	-	10:102532563-103084548	-	NON_PASS	Novel	SUFU, SUFU, MYCN, MAX	Consistent	-	-	-
P2803	CNS	Medulloblastoma (MB)	SUFU	ENST00000369902	Deletion	-	10:99229925-111212987	-	NON_PASS	Novel	SUFU, MAP2K4	Refine (SUFU)	SUFU	-	-
P2623	Liver	Hepatoblastoma (HB)	TERT	ENST00000310581	Substitution	-	5:1295113G>A	-	PASS	Known via SOC	CTNNB1, TERT	Consistent	-	-	-
P2337	Sarcoma	Rhabdomyosarcoma (RMS)	TP53	ENST00000269305	Substitution	Domain_1	17:7674894G>A	c.637C>T p.(Arg213*)	PASS	Novel	NRAS, TP53	Consistent	-	-	-
P3053	Sarcoma	Osteosarcoma (OS)	TP53	ENST00000269305	Deletion	-	17:7680302-7680617	-	PASS	Novel	PDGFRA, TP53, CDKN2A	Consistent	-	Hypermutation (Check	-
P3269	Adrenal	Adrenocortical carcinoma (ACC)	UPD11p	-	UPD	Germline	-	-	PASS	Known via SOC	UPD11p, CTNNB1, GNAS	Consistent	-	-	11p LOH
P2847	CNS	Biphasic neuroepithelial tumour (LGG/HG	ZNF394-BRAF	-	Fusion	-	7:99495253-140783251	-	NON_PASS	Novel	CDKN2A, ZNF394-BRAF	Consistent	-	BRAF	-
