# Supplementary material for: The NHS England 100,000 Genomes Project: feasibility and utility of centralised genome sequencing for children with cancer
Source: Br J Cancer. 2022 Apr 22;127(1):137–44. doi: 10.1038/s41416-022-01788-5 (PMC9276782; doi:10.1038/s41416-022-01788-5)
Supplement: Supplementary file 6 — Supplementary Table 4 [file 41416_2022_1788_MOESM6_ESM.pdf]

Sample	Tissue	Cancer Diagnosis	SOC test	Gene	Tissue origin	Variant type	SOC result
P2058	CNS	Astroblastoma (AB)	FISH	BCOR	Somatic	Rearrangement	Undetected
P2058	CNS	Astroblastoma (AB)	FISH	CIC	Somatic	Rearrangement	Undetected
P2337	Sarcoma	Rhabdomyosarcoma (RMS)	Targeted (MLPA)	TP53	Germline	CNV	Undetected
P2337	Sarcoma	Rhabdomyosarcoma (RMS)	FISH	FOXO1	Somatic	Rearrangement	Undetected
P2337	Sarcoma	Rhabdomyosarcoma (RMS)	FISH	PAX3	Somatic	Rearrangement	Undetected
P2337	Sarcoma	Rhabdomyosarcoma (RMS)	FISH	PAX7	Somatic	Rearrangement	Undetected
P2337	Sarcoma	Rhabdomyosarcoma (RMS)	Targeted (sequencing)	TP53	Germline	SNV/indel	Undetected
P2571	Ovarian	Ovarian granulosa cell tumour (OV GRAN)	Multi-Gene NGS Panel	-	Somatic	SNV/indel	Undetected
P2623	Liver	Hepatoblastoma (HB)	Multi-Gene NGS Panel	-	Somatic	Deletion	Deteted (CTNNB1)
P2623	Liver	Hepatoblastoma (HB)	Multi-Gene NGS Panel	-	Somatic	SNV/indel	Deteted (TERT)
P2624	CNS	Medulloblastoma (MB)	FISH	MYC	Somatic	Amplification	Undetected
P2624	CNS	Medulloblastoma (MB)	FISH	MYCN	Somatic	Amplification	Undetected
P2624	CNS	Medulloblastoma (MB)	Targeted (sequencing)	TP53	Somatic	SNV/indel	Undetected
P2625	Sarcoma	Congenital infantile fibrosarcoma (CIFS)	RT-PCR	ETV6-NTRK3	Somatic	Fusion	Deteted (ETV6-NTRK3)
P2625	Sarcoma	Congenital infantile fibrosarcoma (CIFS)	Multi-Gene NGS Panel	-	Somatic	SNV/indel	Undetected
P2626	Sarcoma	Rhabdomyosarcoma (RMS)	FISH	FOXO1	Somatic	Rearrangement	Undetected
P2627	CNS	Dysembryoplastic neuroepithelial tumour (DNET)	IHC	ATRX	Somatic	Loss	Undetected
P2627	CNS	Dysembryoplastic neuroepithelial tumour (DNET)	IHC	MLH1	Somatic	Loss	Undetected
P2627	CNS	Dysembryoplastic neuroepithelial tumour (DNET)	IHC	MSH2	Somatic	Loss	Undetected
P2627	CNS	Dysembryoplastic neuroepithelial tumour (DNET)	IHC	MSH6	Somatic	Loss	Undetected
P2627	CNS	Dysembryoplastic neuroepithelial tumour (DNET)	IHC	PMS2	Somatic	Loss	Undetected
P2627	CNS	Dysembryoplastic neuroepithelial tumour (DNET)	IHC	BRAF	Somatic	SNV/indel	Undetected
P2627	CNS	Dysembryoplastic neuroepithelial tumour (DNET)	IHC	IDH1	Somatic	SNV/indel	Undetected
P2627	CNS	Dysembryoplastic neuroepithelial tumour (DNET)	IHC	IDH2	Somatic	SNV/indel	Undetected
P2720	Sarcoma	Ewing's sarcoma (ES)	FISH	EWSR1-FLI1	Somatic	Fusion	Deteted (EWSR1-FLI1)
P2720	Sarcoma	Ewing's sarcoma (ES)	Multi-Gene NGS Panel	-	Somatic	SNV/indel	Undetected
P2766	PNS	Neuroblastoma (NB)	FISH	MYCN	Somatic	Amplification	Undetected
P2766	PNS	Neuroblastoma (NB)	FISH	ATM	Somatic	Loss	Undetected
P2766	PNS	Neuroblastoma (NB)	Targeted (sequencing)	ALK	Somatic	SNV/indel	Undetected
P2767	CNS	Pineoblastoma (PB)	IHC	H3F3A	Somatic	SNV/indel	Undetected
P2774	PNS	Neuroblastoma (NB)	FISH	MYCN	Somatic	Amplification	Undetected
P2774	PNS	Neuroblastoma (NB)	FISH	ATM	Somatic	Loss	Undetected
P2774	PNS	Neuroblastoma (NB)	Targeted (sequencing)	ALK	Somatic	SNV/indel	Undetected
P2801	CNS	Medulloblastoma (MB)	FISH	MYC	Somatic	Amplification	Undetected
P2801	CNS	Medulloblastoma (MB)	FISH	MYCN	Somatic	Amplification	Undetected
P2801	CNS	Medulloblastoma (MB)	IHC	SMARCA4	Somatic	Loss	Undetected
P2803	CNS	Medulloblastoma (MB)	FISH	MYC	Somatic	Amplification	Undetected
P2806	CNS	Pilocytic astrocytoma (PA)	Targeted (MLPA)	MLH1	Germline	CNV	Undetected
P2806	CNS	Pilocytic astrocytoma (PA)	Targeted (MLPA)	MSH2	Germline	CNV	Undetected
P2806	CNS	Pilocytic astrocytoma (PA)	Targeted (MLPA)	MSH6	Germline	CNV	Undetected
P2806	CNS	Pilocytic astrocytoma (PA)	Targeted (MLPA)	NF1	Germline	CNV	Undetected
P2806	CNS	Pilocytic astrocytoma (PA)	Targeted (MLPA)	PMS2	Germline	CNV	Undetected
P2806	CNS	Pilocytic astrocytoma (PA)	FISH	KIAA1549-BRAF	Somatic	Fusion	Undetected
P2806	CNS	Pilocytic astrocytoma (PA)	IHC	BRAF	Somatic	SNV/indel	Undetected
P2806	CNS	Pilocytic astrocytoma (PA)	IHC	H3F3A	Somatic	SNV/indel	Undetected
P2806	CNS	Pilocytic astrocytoma (PA)	Targeted (sequencing)	NF1	Germline	SNV/indel	Deteted (NF1)
P2806	CNS	Pilocytic astrocytoma (PA)	Targeted (sequencing)	MLH1	Germline	SNV/indel	Undetected
P2806	CNS	Pilocytic astrocytoma (PA)	Targeted (sequencing)	MSH2	Germline	SNV/indel	Undetected
P2806	CNS	Pilocytic astrocytoma (PA)	Targeted (sequencing)	MSH6	Germline	SNV/indel	Undetected
P2806	CNS	Pilocytic astrocytoma (PA)	Targeted (sequencing)	PMS2	Germline	SNV/indel	Undetected
P2830	CNS	Glioma with molecular features of pleomorphic xanthoastro	FISH	KIAA1549-BRAF	Somatic	Fusion	Undetected
P2830	CNS	Glioma with molecular features of pleomorphic xanthoastro	IHC	IDH1	Somatic	SNV/indel	Undetected
P2831	CNS	Pilocytic astrocytoma (PA)	FISH	KIAA1549-BRAF	Somatic	Fusion	Deteted (KIAA1549-BRAF)
P2831	CNS	Pilocytic astrocytoma (PA)	IHC	BRAF	Somatic	SNV/indel	Undetected
P2831	CNS	Pilocytic astrocytoma (PA)	IHC	H3F3A	Somatic	SNV/indel	Undetected
P2847	CNS	Biphasic neuroepithelial tumour (LGG/HGG)	IHC	CDK4	Somatic	Amplification	Undetected
P2847	CNS	Biphasic neuroepithelial tumour (LGG/HGG)	IHC	EGFR	Somatic	Amplification	Undetected
P2847	CNS	Biphasic neuroepithelial tumour (LGG/HGG)	IHC	MYCN	Somatic	Amplification	Undetected
P2847	CNS	Biphasic neuroepithelial tumour (LGG/HGG)	IHC	PDGFRA	Somatic	Amplification	Undetected
P2847	CNS	Biphasic neuroepithelial tumour (LGG/HGG)	FISH	KIAA1549-BRAF	Somatic	Fusion	Undetected
P2847	CNS	Biphasic neuroepithelial tumour (LGG/HGG)	IHC	BRAF	Somatic	SNV/indel	Undetected
P2847	CNS	Biphasic neuroepithelial tumour (LGG/HGG)	IHC	H3F3A	Somatic	SNV/indel	Undetected
P2878	Sarcoma	Rhabdomyosarcoma (RMS)	IHC	ALK	Somatic	Amplification	Undetected
P2878	Sarcoma	Rhabdomyosarcoma (RMS)	FISH	FOXO1	Somatic	Rearrangement	Undetected
P2878	Sarcoma	Rhabdomyosarcoma (RMS)	FISH	PAX3	Somatic	Rearrangement	Undetected
P2878	Sarcoma	Rhabdomyosarcoma (RMS)	FISH	PAX7	Somatic	Rearrangement	Undetected
P2887	CNS	Medulloblastoma (MB)	FISH	MYC	Somatic	Amplification	Undetected
P2887	CNS	Medulloblastoma (MB)	FISH	MYCN	Somatic	Amplification	Undetected
P2887	CNS	Medulloblastoma (MB)	IHC	SMARCA4	Somatic	Loss	Undetected
P2955	CNS	Anaplastic ependymoma (EP)	Targeted (MLPA)	NF1	Germline	CNV	Undetected
P2955	CNS	Anaplastic ependymoma (EP)	Targeted (MLPA)	SPRED1	Germline	CNV	Undetected
P2955	CNS	Anaplastic ependymoma (EP)	IHC	PMS2	Somatic	Loss	Deteted (PMS2)
P2955	CNS	Anaplastic ependymoma (EP)	IHC	MLH1	Somatic	Loss	Undetected
P2955	CNS	Anaplastic ependymoma (EP)	IHC	MSH2	Somatic	Loss	Undetected
P2955	CNS	Anaplastic ependymoma (EP)	IHC	MSH6	Somatic	Loss	Undetected
P2955	CNS	Anaplastic ependymoma (EP)	Targeted (sequencing)	GNAS	Germline	SNV/indel	Undetected
P2955	CNS	Anaplastic ependymoma (EP)	Targeted (sequencing)	NF1	Germline	SNV/indel	Undetected
P2955	CNS	Anaplastic ependymoma (EP)	Targeted (sequencing)	SPRED1	Germline	SNV/indel	Undetected
P2981	CNS	Medulloblastoma (MB)	FISH	MYC	Somatic	Amplification	Undetected
P2981	CNS	Medulloblastoma (MB)	FISH	MYCN	Somatic	Amplification	Undetected
P2981	CNS	Medulloblastoma (MB)	IHC	SMARCA4	Somatic	Loss	Undetected
P2981	CNS	Medulloblastoma (MB)	IHC	H3F3A	Somatic	SNV/indel	Undetected
P2994	Renal	Renal cell carcinoma (RCC)	FISH	SS18	Somatic	Rearrangement	Undetected
P2994	Renal	Renal cell carcinoma (RCC)	Multi-Gene NGS Panel	-	Somatic	SNV/indel	Deteted (CTNNB1)
P2994	Renal	Renal cell carcinoma (RCC)	Multi-Gene NGS Panel	-	Somatic	SNV/indel	Deteted (KRAS)
P2994	Renal	Renal cell carcinoma (RCC)	Multi-Gene NGS Panel	-	Germline	SNV/Indel/CNV	Undetected
P2994	Renal	Renal cell carcinoma (RCC)	Targeted (MS-MLPA)	UPD11p	Germline	UPD	Undetected
P3038	Liver	Hepatoblastoma (HB)	Targeted (MLPA)	APC	Germline	CNV	Undetected
P3038	Liver	Hepatoblastoma (HB)	Targeted (sequencing)	APC	Germline	SNV/indel	Undetected
P3053	Sarcoma	Osteosarcoma (OS)	Multi-Gene NGS Panel	-	Somatic	Amplification	Deteted (PDGFRA)
P3072	Renal	Wilms' tumour (WT)	Multi-Gene NGS Panel	-	Somatic	SNV/indel	Undetected
P3088	CNS	Diffuse Leptomeningeal Glioneuronal Tumour (DLGNT)	FISH	KIAA1549-BRAF	Somatic	Fusion	Undetected
P3088	CNS	Diffuse Leptomeningeal Glioneuronal Tumour (DLGNT)	IHC	BRAF	Somatic	SNV/indel	Undetected
P3088	CNS	Diffuse Leptomeningeal Glioneuronal Tumour (DLGNT)	IHC	IDH1	Somatic	SNV/indel	Undetected
P3089	PNS	Ganglio-neuroblastoma (G-NB)	FISH	MYCN	Somatic	Amplification	Undetected
P3089	PNS	Ganglio-neuroblastoma (G-NB)	FISH	ATM	Somatic	Loss	Undetected
P3091	Renal	Wilms' tumour (WT)	-	-	-	-	No previous testing
P3094	Lymphatic	Lymphoma (LYM)	FISH	CDKN2A	Somatic	Deletion	Deteted (CDKN2A)
P3094	Lymphatic	Lymphoma (LYM)	FISH	BCR-ABL1	Somatic	Fusion	Undetected
P3094	Lymphatic	Lymphoma (LYM)	FISH	ETV6-RUNX1	Somatic	Fusion	Undetected
P3094	Lymphatic	Lymphoma (LYM)	FISH	BCL2	Somatic	Rearrangement	Undetected
P3094	Lymphatic	Lymphoma (LYM)	FISH	BCL6	Somatic	Rearrangement	Undetected
P3094	Lymphatic	Lymphoma (LYM)	FISH	IGH	Somatic	Rearrangement	Undetected
P3094	Lymphatic	Lymphoma (LYM)	FISH	MLL	Somatic	Rearrangement	Undetected
P3094	Lymphatic	Lymphoma (LYM)	FISH	MYC	Somatic	Rearrangement	Undetected
P3153	Sarcoma	Undifferentiated sarcoma (US)	-	-	-	-	No previous testing
P3155	Liver	Hepatoblastoma (HB)	-	-	-	-	No previous testing
P3221	Teratoma	Immature teratoma (IT)	-	-	-	-	No previous testing
P3244	Liver	Hepatoblastoma (HB)	Chromosome breakage stu	-	Germline	Cyto	Undetected
P3269	Adrenal	Adrenocortical carcinoma (ACC)	Multi-Gene NGS Panel	-	Somatic	SNV/indel	Deteted (CTNNB1)
P3269	Adrenal	Adrenocortical carcinoma (ACC)	Multi-Gene NGS Panel	-	Somatic	SNV/indel	Deteted (GNAS)
P3269	Adrenal	Adrenocortical carcinoma (ACC)	Targeted (MS-MLPA)	UPD11p	Germline	UPD	Deteted (UPD11p)
P3311	Adrenal	Adrenocortical carcinoma (ACC)	IHC	MLH1	Somatic	Loss	Undetected
P3311	Adrenal	Adrenocortical carcinoma (ACC)	IHC	MSH2	Somatic	Loss	Undetected
P3311	Adrenal	Adrenocortical carcinoma (ACC)	IHC	MSH6	Somatic	Loss	Undetected
P3311	Adrenal	Adrenocortical carcinoma (ACC)	IHC	PMS2	Somatic	Loss	Undetected
P3311	Adrenal	Adrenocortical carcinoma (ACC)	Multi-Gene NGS Panel	-	Somatic	SNV/indel	Deteted (CTNNB1)
